# Metabolic engineering of *Escherichia coli* for efficient production of L-5-hydroxytryptophan from glucose

**DOI:** 10.1186/s12934-022-01920-3

**Published:** 2022-09-24

**Authors:** Zhen Zhang, Zichen Yu, Jinduo Wang, Yifa Yu, Lanxiao Li, Pengjie Sun, Xiaoguang Fan, Qingyang Xu

**Affiliations:** 1 College of Biotechnology, Tianjin University of Science & Technology, Tianjin, 300457 People’s Republic of China; 2 Nanning Harworld Biological Technology Co., Ltd, Nanning, 530000 People’s Republic of China; 3 Key Laboratory of Industrial Fermentation Microbiology, Ministry of Education, Tianjin University of Science & Technology, Tianjin, 300457 People’s Republic of China

**Keywords:** L-5-hydroxytryptophan, *Escherichia coli*, Systematic modular engineering, Human TPH2 mutant, NA(D)PH regeneration

## Abstract

**Background:**

5-hydroxytryptophan (5-HTP), the direct biosynthetic precursor of the neurotransmitter 5-hydroxytryptamine, has been shown to have unique efficacy in the treatment of a variety of disorders, including depression, insomnia, and chronic headaches, and is one of the most commercially valuable amino acid derivatives. However, microbial fermentation for 5-HTP production continues to face many challenges, including low titer/yield and the presence of the intermediate L-tryptophan (L-Trp), owing to the complexity and low activity of heterologous expression in prokaryotes. Therefore, there is a need to construct an efficient microbial cell factory for 5-HTP production.

**Results:**

We describe the systematic modular engineering of wild-type *Escherichia coli* for the efficient fermentation of 5-HTP from glucose. First, a xylose-induced T7 RNA polymerase-P_*T7*_ promoter system was constructed to ensure the efficient expression of each key heterologous pathway in *E. coli*. Next, a new tryptophan hydroxylase mutant was used to construct an efficient tryptophan hydroxylation module, and the cofactor tetrahydrobiopterin synthesis and regeneration pathway was expressed in combination. The L-Trp synthesis module was constructed by modifying the key metabolic nodes of tryptophan biosynthesis, and the heterologous synthesis of 5-HTP was achieved. Finally, the NAD(P)H regeneration module was constructed by the moderate expression of the heterologous GDH_*esi*_ pathway, which successfully reduced the surplus of the intermediate L-Trp. The final engineered strain HTP11 was able to produce 8.58 g/L 5-HTP in a 5-L bioreactor with a yield of 0.095 g/g glucose and a maximum real-time productivity of 0.48 g/L/h, the highest values reported by microbial fermentation.

**Conclusion:**

In this study, we demonstrate the successful design of a cell factory for high-level 5-HTP production, combined with simple processes that have potential for use in industrial applications in the future. Thus, this study provides a reference for the production of high-value amino acid derivatives using a systematic modular engineering strategy and a basis for an efficient engineered strain development of 5-HTP high-value derivatives.

**Supplementary Information:**

The online version contains supplementary material available at 10.1186/s12934-022-01920-3.

## Background

L-5-hydroxytryptophan (5-HTP) is a non-protein amino acid and a precursor of several important physiological functions, including the neurotransmitter serotonin (also known as 5-hydroxytryptamine, 5-HT), and the hormone melatonin [1]. At present, the application of 5-HTP in medication and health care is expanding, and its commercial potential continues to be explored due to its remarkable efficacy in treating several disorders and diseases, such as depression, insomnia, Parkinson’s syndrome, and Alzheimer’s disease (Additional file 1: Fig. S1b) [2, 3].

Currently, the mainstream commercial production of 5-HTP continues to be derived from plant seeds; however, the availability of the raw material *Griffonia simplifolia* seeds is seasonal and regional, resulting in limited supply and high market prices [4]. In addition, although the chemical synthesis of 5-HTP has been attempted [5], it is not economically feasible due to the complex process and high substrate cost [1, 4]. As traditional production methods can no longer meet the growing market demand, a more practical method is needed to provide higher and more stable production capacity. As an efficient, low-cost, sustainable, and environmental-friendly production technology, microbial fermentation has been applied to the large-scale industrial manufacture of several valuable natural molecules [6, 7] and is a viable alternative for the production of 5-HTP. Among the organisms used for this method, *Escherichia coli*, the model strain of prokaryotes, has the advantages of a clear genetic background and simple culture method [8, 9], which make it an ideal candidate for the 5-HTP microbial synthesis of chassis cells [10–12].

**Fig. 1:**
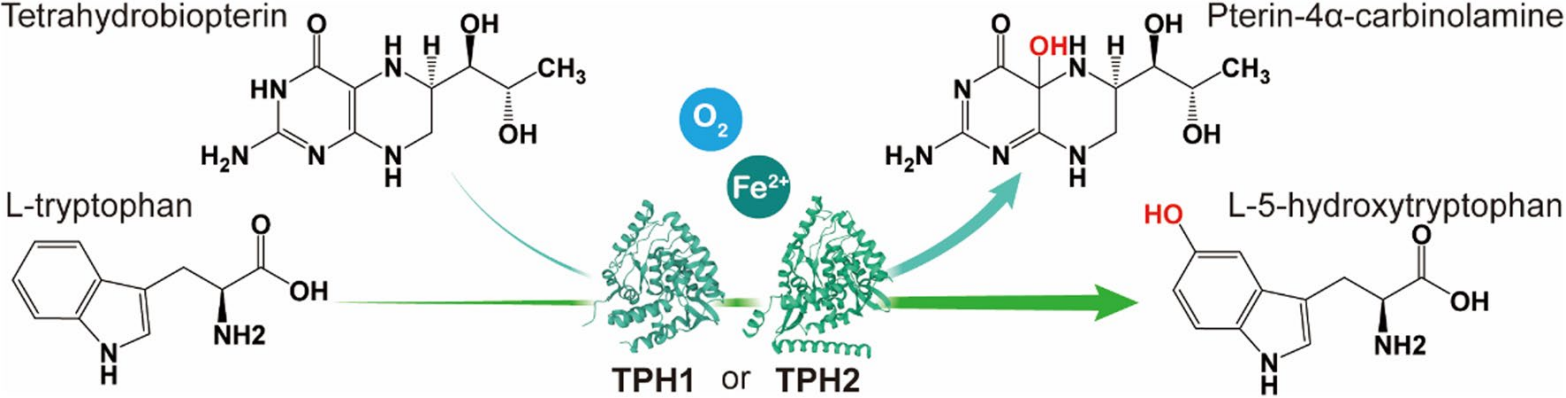
The tetrahydrobiopterin-dependent tryptophan hydroxylase pathway

In mammals, oxygen and L-tryptophan (L-Trp) are used as substrates for the biosynthesis of 5-HTP catalyzed by tryptophan hydroxylase (TPH), with Fe^2+^ and tetrahydrobiopterin (BH4) as cofactors [13, 14] (Fig. 1), whereas a similar natural pathway has not been reported in prokaryotes. The absence of the prokaryotic tryptophan hydroxylase pathway poses great difficulties for 5-HTP synthesis in *E. coli*, mostly because of the complexity and low activity of the heterologous pathway expressed in *E. coli*. Therefore, some researchers have addressed these challenges by circumventing the natural tryptophan hydroxylation pathway. Sun et al. [15] devised a distinctive 5-HTP biosynthetic route that first converts the unnatural substrate anthranilate into 5-hydroxyanthranilate (5-HAA) using a novel salicylate 5-hydroxylase, and then transforms 5-HAA to 5-HTP using the *E. coli* endogenous tryptophan synthesis pathway. The final 5-HTP yield of this method reached 98.09 mg/L with a by-product L-Trp content of 23.05 mg/L, and no accumulation of intermediate 5-HAA was detected. Lin [16] established a phenylalanine 4‑hydroxylase (P4H)-mediated tryptophan hydroxylation pathway by shifting the substrate preference of P4H and simultaneously introducing a heterologous tetrahydromonapterin (MH4) cycling mechanism. On this basis, the engineered *E. coli* could utilize endogenous MH4 as a coenzyme to complete the L-Trp hydroxylation reaction; ultimately, ~ 1.2 g/L 5-HTP was produced with 2 g/L L-Trp as substrate after 48 h of shake flask fermentation. In another study [17], similar to the above strategy, an aromatic amino acid hydroxylase (CtAAAH) from *Cupriavidus taiwanensis* was heterologously expressed in *E. coli* and its L-Trp hydroxylation activity was optimized by protein engineering to obtain a 5-HTP titer of 0.96 g/L. In addition, Hara [11] introduced *Bacillus subtilis* glucose dehydrogenase (encoded by the *gdh*_*bus*_ gene) into recombinant *E. coli*, which indirectly promoted the regeneration of BH4 and enhanced the intracellular tryptophan hydroxylation reaction catalyzed by phenylalanine hydroxylase, resulting in the synthesis of 0.55 g/L 5-HTP. It is obvious that researchers have made great efforts to achieve efficient heterologous synthesis of 5-HTP using various advanced tools including synthetic biology and protein engineering. However, most of their findings remain far from practical application.

Until Wang et al. [12] successfully expressed the BH4-dependent human TPH2 pathway heterologously in *E. coli* BL21 and obtained a high titer of 5-HTP (5.1 g/L, in a 10 L bioreactor), it was possible to the industrialize production of 5-HTP by microbial fermentation. In their study, the BH4 biosynthesis and regeneration pathways from different species were screened and reconstituted, and a truncated form of the *TPH2* gene was introduced to enhance the intracellular hydroxylation reaction, followed by the construction of an endogenous L-Trp pathway to achieve the efficient synthesis of 5-HTP with glycerol as the carbon source. Nevertheless, the process suffers from serious L-Trp spillage (> 8 g/L), which hinders downstream extraction and is unfavorable for commercial scale-up production.

**Fig. 2:**
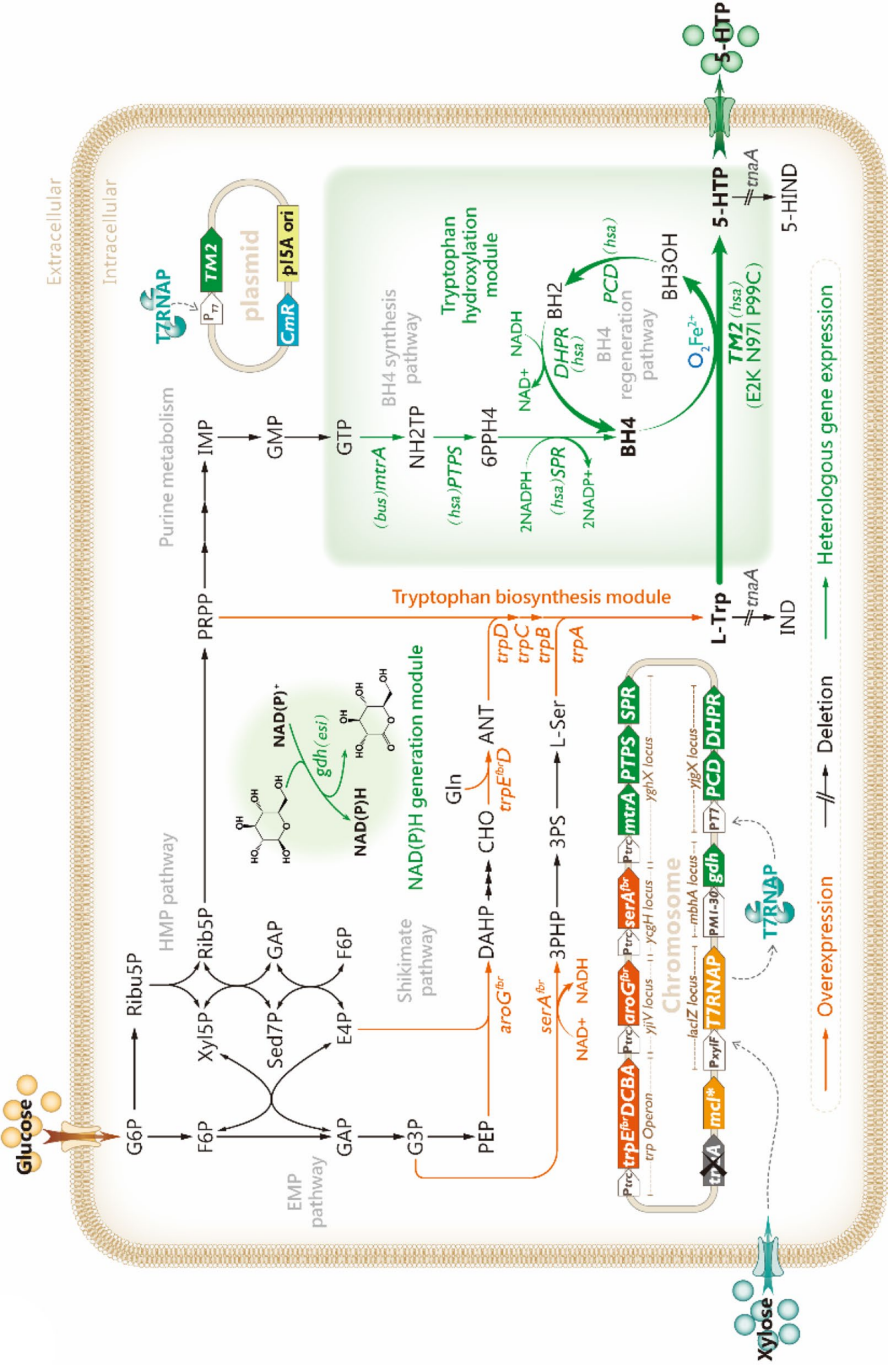
Metabolic pathway designs and key metabolic engineering strategies for 5-HTP production in *E. coli*. The orange arrows indicate the overexpression of relevant endogenous genes in the chassis cells through chromosome integration or promoter replacement. The black arrows with “**//**” indicate the deletion of the relevant genes. The green arrows indicate the introduction of the relevant heterologous genes. Three arrows in series indicate a multi-step reaction

Considering the economic viability of large-scale 5-HTP fermentation, efficient microbial cell factories that can stably achieve high titers, yields, and productivity of 5-HTP synthesis with low L-Trp residues are particularly desirable. Hence, in this study, systematic modular engineering (Fig. 2) was used to construct a 5-HTP biosynthetic pathway in *E. coli* K-12 w3110 to achieve a high-level production of 5-HTP. First, a “xylose-induced T7 RNA polymerase-PT7 promoter system” [18–20] was constructed to ensure the efficient and controlled expression of each heterologous gene. Next, BH4 synthesis and regeneration pathways were combined and optimized, and a human TPH2 mutant with higher heterologous hydroxylation activity [21] was selected to build a stable tryptophan hydroxylation module. Then, the precursor L-Trp pool was enriched to achieve the heterologous synthesis of 5-HTP. Finally, the NAD(P)H regeneration module was constructed by moderate expression of glucose dehydrogenase (GDH_*esi*_) derived from *Exiguobacterdium sibiricum*, which restored intracellular redox homeostasis and substantially reduced the surplus of the intermediate L-Trp. Based on the efficient fermentation strategy established in this study, the resultant strain could produce 5-HTP 8.58 g/L in a 5-L bioreactor with a yield of 0.095 g/g glucose and a maximum real-time productivity of 0.48 g/L/h. This study provides a cost-effective 5-HTP fermentation process that achieves the highest titer and yield with a low by-product L-Trp residue of 5-HTP production by microbial fermentation.

## Results

### Construction of L-tryptophan hydroxylation module

The heterologous expression of mammalian genes in *E. coli* often requires higher expression levels because of the lower stability and solubility of their translation products [12]. However, the “xylose-inducible T7 RNA polymerase-P_*T7*_ promoter system” satisfied this requirement [19, 22]. Hence, a P_*xylF*_-driven T7 RNA polymerase gene was first integrated into the *lacIZ* locus of *E. coli* K-12 w3110, and the promoter region of the *mlc* gene, encoding a transcriptional regulator of PTS genes, was subsequently mutated to alleviate carbon catabolite repression [18, 20]. In addition, the native *tnaA* gene was deleted to prevent the degradation of L-Trp and 5-HTP (Fig. 2). These modifications resulted in chassis HTP01.

**Fig. 3:**
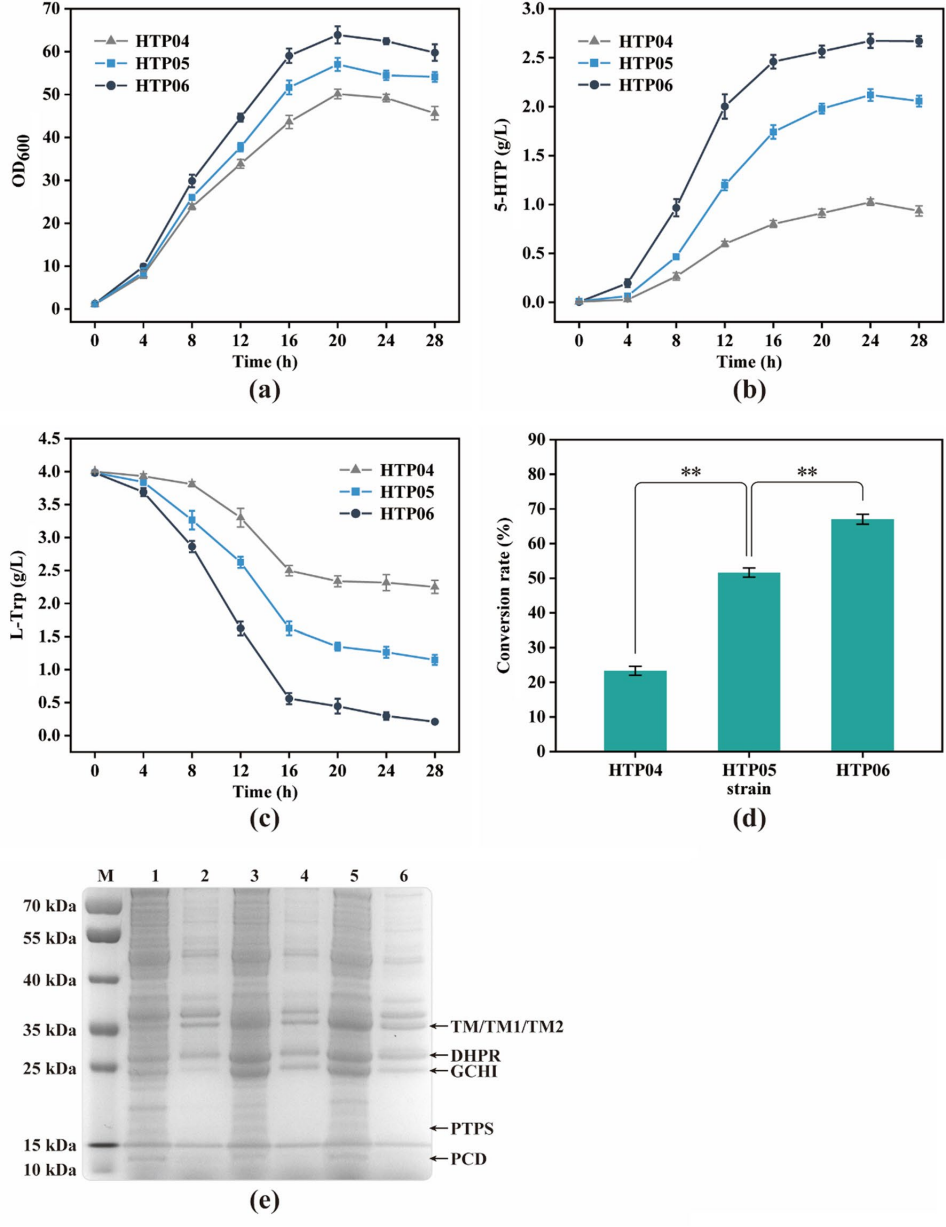
Whole-cell bioconversion of L-tryptophan into 5-HTP using HTP04-06 strains. **a** Cell growth. **b** 5-HTP production. **c** L-Trp consumption. **d** The ultimate conversion rate of L-Trp to 5-HTP. **e** Protein expression in HTP04-HTP06 strains after 12-h cultivation. M: mark; 1, 2: Soluble and inclusion expression of HTP04; 3, 4: Soluble and inclusion expression of HTP05; 5, 6: Soluble and inclusion expression of HTP06. The genotype of strains: HTP04, *E. coli* W3110 *∆tnaA lacI*::P_*xylF*_-*T7RNAP mlc*::mlc* *yghX*::P_*trc*_-*mtrA*-*PTPS*-*SPR yjgX*::P_*T7*_-*PCD*-*DHPR* pSTV-TM; HTP05, HTP04 pSTV-TM::pSTV-TM1; HTP06, HTP04 pSTV-TM::pSTV-TM2. The data are presented as averages, and the error bars represent standard deviations (n = 3 independent experiments). ** P < 0.01

The continuous availability of the cofactor BH4 is necessary for TPH-mediated tryptophan hydroxylation reactions, and exogenous supplementation with expensive BH4 is impractical. Therefore, the ability of engineered strains to synthesize BH4 is crucial for practical applications, which is also the first issue to be addressed for 5-HTP heterologous synthesis by microorganisms [4]. Further, the BH4 regeneration pathway, which requires high-intensity expression [12], is necessary to prevent the generation of competitive inhibitors against BH4 [23–26], such as 7-substituted pterins and BH2, ensuring a sufficient supply of reduced BH4. Therefore, relevant gene routes were rationally designed and modified. To this end, the three key genes of the BH4 synthesis pathway, *mtrA* (encoding GTP cyclohydrolase I, GCHI), *PTPS* (encoding 6-pyruvate-tetrahydropterin synthase, PTPS) and *SPR *(encoding sepiapterin reductase, SPR), were tandemly linked and driven by the trc promoter to form a mini-operon that was integrated into the *yghX* locus of the HTP01 genome, resulting in strain HTP02. Similarly, the two key genes of the BH4 regeneration pathway, *PCD* (encoding pterin-4α-carbinolamine dehydratase, PCD) and *DHPR *(encoding dihydropteridine reductase, DHPR), were then connected in series and driven by a strong T7 promoter to form another mini-operon, which was integrated into the *yjgX* locus of the HTP02 genome to obtain the strain HTP03. Meanwhile, 5-untranslated regions containing strong ribosome-binding sites (AAGGAG) were inserted in front of *PTPS*, *SPR*, and *DHPR*, respectively, to ensure adequate translation [12, 27].

The solubility and catalytic activity of tryptophan hydroxylase in *E. coli* are crucial for the heterologous synthesis of 5-HTP, and researchers have made numerous efforts to screen and exploit high-activity hydroxylases. In this study, the truncated form of human TPH2, TM (TPH2 mutant with a deletion of the first 145 N-terminal and 24 C-terminal amino acids [12]), and its two mutants, TM1 (with a mutated amino acid E2K [28]) and TM2 (with mutated amino acids E2K, N97I, and P99C [21]), were selected for the test of 5-HTP heterologous synthesis in *E. coli*. The mutant genes *TM*, *TM1*, and *TM2* under the control of the T7 promoter were inserted into plasmid pSTV28 to construct the overexpression plasmids pSTV-TM, pSTV-TM1, and pSTV-TM2. Then, the three plasmids were introduced into HTP03 by electroporation, resulting in HTP04, HTP05, and HTP06 strains. Subsequent SDS-PAGE analysis (Fig. 3e) showed that all the heterologous proteins, except SPR, showed clear bands on the gel, and soluble expression occupied a greater proportion. Meanwhile, the expression levels of both TM1 and TM2 were significantly higher than that of TM. Furthermore, the ability of HTP04/HTP05/HTP06 strains to facilitate the whole-cell synthesis of 5-HTP was evaluated using enriched media at the shake flask fermentation scale with exogenous supplementation of 4 g/L L-Trp as the substrate. The results indicated that after a 28-h period of cultivation, HTP06 produced the highest titer of 5-HTP (2.67 g/L), which was 25.94% and 161.76% higher than the titer of HTP05 (2.12 g/L) and HTP04 (1.02 g/L), respectively (Fig. 3b), with a final conversion rate from L-Trp to 5-HTP as high as 67.02% (Fig. 3d). The 5-HTP titer per unit cell mass of HTP06 reached 0.127 g/g_CDW_, which was 17.59% and 118.97% higher than that of HTP05 (0.108 g/g_CDW_) and HTP04 (0.058 g/g_CDW_), respectively (Fig.S4). Meanwhile, the consumption of L-Trp (Fig. 3c) also verified these results, suggesting that the uptake of L-Trp and the secretion of 5-HTP in the cells were fluent. In addition, the biomass (OD_600_) of HTP06 was the highest (63.91), and it maintained a higher growth rate during the logarithmic growth period (Fig. 3a). These results demonstrate that the heterologous tryptophan hydroxylation module of *E. coli* designed in this study can operate efficiently and achieve rapid intracellular conversion of L-Trp to 5-HTP without the exogenous addition of BH4. Moreover, the strain HTP06 (containing the *TM2* gene) showed the highest 5-HTP production per cell mass and biomass under the same fermentation conditions, indicating that the selected hydroxylase mutant TM2 has superior L-Trp hydroxylation activity and less cytotoxicity, which is more suitable for heterologous expression in *E. coli* for the synthesis of 5-HTP, consistent with previous research findings [21].

### Construction of tryptophan biosynthesis module

The precursor L-Trp pool needs to be enriched to enable the engineered strain to synthesize 5-HTP from scratch using a simple carbon source, while relief feedback inhibition and the overexpression of rate-limiting enzymes at key metabolic nodes have been shown to be important and effective strategies for the overproduction of L-Trp [29].

**Fig. 4:**
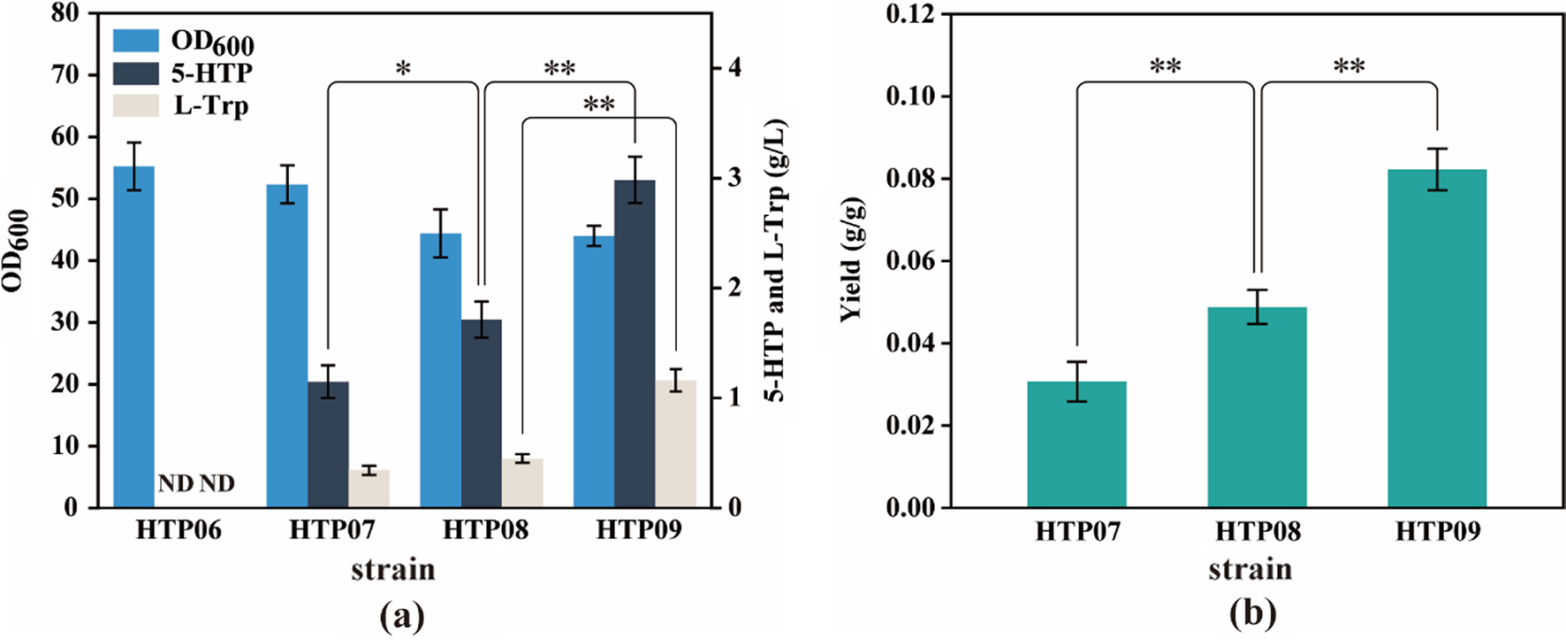
Effect of enriching the precursor L-Trp pool on 5-HTP production. **a** Cell growth, 5-HTP production and L-Trp accumulation of HTP06-09 strains after 26-h period of cultivation in shake flasks with glucose as carbon source. **b** Yield of HTP07-09 strains. The genotype of strains: HTP07, HTP06 *trpLE*::P_*trc*_-*trpE*^*fbr*^; HTP08, HTP07 *yjiV*::P_*trc*_-*aroG*^*fbr*^; HTP09, HTP08 *ycgH*::P_*trc*_-*serA*^*fbr*^. The data are presented as averages, and the error bars represent standard deviations (n = 3 independent experiments). * 0.01 < P < 0.05, ** P < 0.01. ND, not detected

In *E. coli*, the carbon flow is triaged to three aromatic amino acids after passing through the chorismate (CHO) node, and the multi-enzyme system encoded by the tryptophan operon catalyzes the conversion of CHO to tryptophan (Fig. 2). Therefore, ensuring the high expression of the tryptophan operon is essential to enable the carbon flux to flow into the tryptophan synthesis pathway and prevent excessive accumulation of other aromatic amino acids [30]. Here, a trc promoter-driven feedback resistance gene, *trpE*_*S40F*_ [31] (hereafter denoted as t*rpE*^*fbr*^), was used to replace the *trpE* gene, promoter, and propeptide (trpL) sequence of the endogenous tryptophan operon for HTP06 in order to deregulate the negative feedback of L-Trp on the major rate-limiting enzyme anthranilate synthetase and to enhance the expression of the endogenous tryptophan manipulator, resulting in HTP07. The shake flask fermentation results (Fig. 4) showed that strain HTP07 had a 5-HTP titer of 1.15 g/L and a yield of 0.031 5- HTP g/g glucose, while 0.34 g/L of L-Trp accumulation was detected. In contrast, 5-HTP and L-Trp were not detected in the fermentation broth of control strain HTP06. Furthermore, the OD_600_ of HTP07 (52.33) was slightly lower than that of HTP06 (55.24), indicating the limited effects of the relevant modifications to the tryptophan operon on cell growth.

The reaction of 3-deoxy-D-arabino-heptulosonate-7-phosphate (DAHP) synthase, which catalyzes the condensation of phosphoenolpyruvate (PEP) and erythrose-4-phosphate (E4P) to form DAHP, is another key metabolic node in the metabolic network of L-Trp biosynthesis (Fig. 2), as DAHP synthase is one of the rate-limiting enzymes of the pathway [32]. The three isozymes of this enzyme are encoded by the *aroG*, *aroF*, and *aroH* genes, with the *aroG* gene-encoded isozyme accounting for ~ 79% of the overall enzymatic activity [30]. Therefore, to efficiently direct PEP and E4P into the L-Trp biosynthetic pathway, the feedback resistance gene *aroG*_*S180F*_ [33] (hereafter denoted as *aroG*^*fbr*^) driven by the trc promoter was integrated into the *yjiV* locus of the HTP07 genome, resulting in HTP08. Somewhat expectedly, the overexpression of the *aroG*^*fbr*^ gene promoted 5-HTP synthesis with a 48.7% increase in the 5-HTP titer (1.71 g/L) and a 58.06% increase in yield (0.049 5-HTP g/g glucose) for HTP08 compared with HTP07 (Fig. 4). However, it should also be noted that the OD_600_ of HTP08 was maintained at 44.4, which was 15.15% lower than that of HTP07, indicating that the modification had a greater negative impact on cell growth. In addition, 0.45 g/L of L-Trp was still detected in the fermentation broth of HTP08.

The condensation of indole and L-serine (L-Ser) is the terminus of the tryptophan biosynthesis pathway (Fig. 2), and a sufficient supply of L-Ser facilitates the synthesis of L-Trp [27]. However, the rate-limiting enzyme 3-phosphoglycerate dehydrogenase (PGDH, encoded by *serA*) of the L-Ser synthesis pathway is feedback-inhibited by L-Ser. Thus, a trc promoter-controlled feedback resistance gene, *serA*_*H344A/N364A*_ [34] (hereafter denoted as *serA*^*fbr*^), was integrated into the *ycgH* pseudogene locus of HTP08, resulting in the strain HTP09. Surprisingly, the enhanced expression of *serA*^*fbr*^ contributed greatly to the overproduction of 5-HTP, with an increase of 74.27% in the 5-HTP titer (2.98 g/L) and 67.35% in yield (0.082 g/g glucose) for HTP09 compared with HTP08 (Fig. 4). Meanwhile, no significant difference was observed in the OD_600_ of the two engineered strains, indicating that the cell growth was not affected. However, the accumulation of L-Trp in HTP09 increased by 159.42% to 1.16 g/L compared to HTP08.

These experimental results suggest that the deregulation of the negative feedback of key metabolic nodes in the L-Trp biosynthetic pathway at the transcription level can effectively enrich the precursor L-Trp pool, which in turn enables the overproduction of 5-HTP. However, it is worth noting that the highest performing engineered strain HTP09 still has the issue of intermediate L-Trp surplus in the fermentation, which not only wastes the carbon source but also renders product isolation and purification to be more challenging.

### Construction of NAD(P)H generation module

**Fig. 5:**
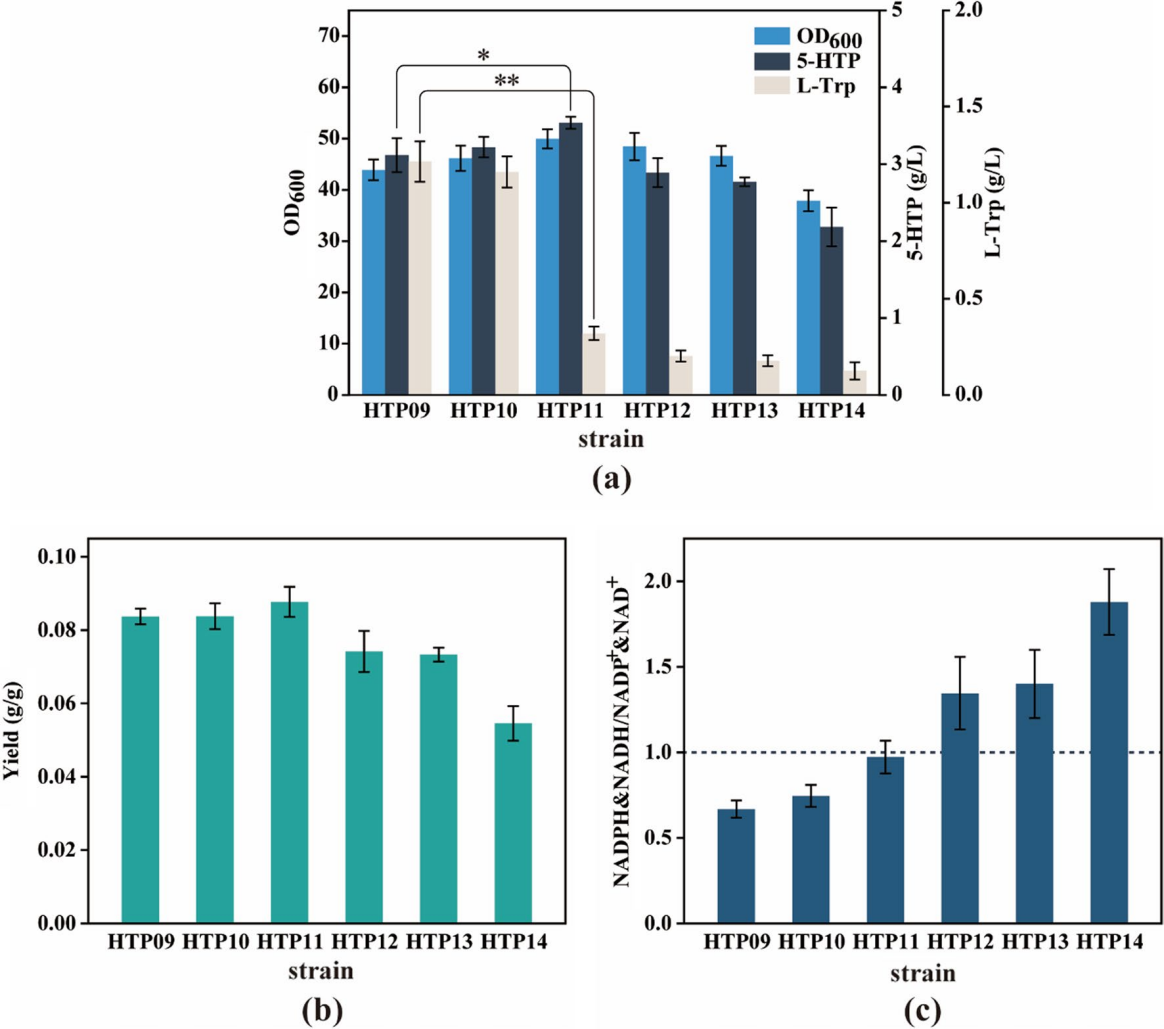
5-HTP production and intracellular NAD(P)H availability in HTP09-HTP14 strains with different promoters of *gdh*_*esi*_. **a** Cell growth, 5-HTP production, and L-Trp accumulation. **b** Yield. **c** Intracellular NAD(P)H/NAD(P)^+^ ratio. The dashed line indicates that the ratio is one. The genotype of strains: HTP10, HTP09 *mbhA*::P_*M1−12*_-*gdh*_*esi*_; HTP11, HTP09, *mbhA*::P_*M1−30*_-*gdh*_*esi*_; HTP12, HTP09 *mbhA*::P_*M1−46*_-*gdh*_*esi*_; HTP13, HTP09 *mbhA*::P_*M1−37*_-*gdh*_*esi*_; HTP14, HTP09 *mbhA*::P_*M1−93*_-*gdh*_*esi*_. The data are presented as averages, and the error bars represent standard deviations (n = 3 independent experiments). * 0.01 < P < 0.05, ** P < 0.01

In the synthetic biology strategy of this study, the heterologous BH4 synthesis and regeneration pathways were NADPH-dependent and NADH-dependent, respectively (Fig. 2). Engineered bacteria require two NADPH molecules for the synthesis of one BH4 molecule from 6PPH4 and one NADH molecule for the regeneration of one BH4 molecule. Consequently, the abundance of NAD(P)H is a crucial factor in 5-HTP overproduction. At the same time, glucose dehydrogenase (GDH) can reduce NAD(P)^+^ to NAD(P)H with glucose as substrate and produces the by-product gluconolactone. And the strategy of regulating intracellular NAD(P)H levels by introducing a GDH-mediated cofactor regeneration pathway to promote target product synthesis and reduce by-product accumulation has proven to be simple and effective [11, 35]. Therefore, we introduced the glucose dehydrogenase (GDH_*esi*_, encoded by *gdh*_*esi*_ gene) pathway derived from *Exiguobacterdium sibiricum*, which can be efficiently expressed in *E. coli* [36], into HTP09 to enhance the NAD(P)H pool. In addition, considering that fluctuations in the relative intracellular NAD(P)H and NAD(P)^+^ contents can directly affect redox homeostasis, which in turn influences energy metabolism, carbon flux allocation, and cell growth [37], the intensity of *gdh*_*esi*_ expression requires delicate regulation to achieve a stable redox state. To this end, *gdh*_*esi*_ was driven by five promoters of different strength (Table 1) [38] that were separately integrated into the *mbhA* locus of HTP09, resulting in HTP10-HTP14 strains. After shake flask fermentation, HTP11 carrying the P_*M1−30*_-*gdh*_*esi*_ gene was found to achieve the highest 5-HTP titer (3.54 g/L) and yield (0.88 g/g glucose), with an increase of 18.79% and 7.32% compared with HTP09, respectively (Fig. 5a and b). Notably, the L-Trp residue of HTP11 was only 0.32 g/L, which was 72.41% lower than that of HTP09. In addition, the cell growth of HTP11 was restored, with the final OD_600_ stabilizing at 49.96. Furthermore, the intracellular availability of reduced cofactors was examined for each engineered strain, and, as expected, the percentage of total reduced cofactors was significantly higher for HTP11 than for HTP09 (Fig. 5c and S5). And, of note, the NAD(P)H/NAD(P)^+^ value of HTP11 was closest to one among all tested strains (Fig. 5c), suggesting that the redox balance is important for the overproduction of 5-HTP. These results indicate that the moderate expression of *gdh*_*esi*_ could effectively increase the intracellular NAD(P)H level and restore redox balance in engineered strains. As a result, the synthesis and regeneration of BH4 were promoted, leading to a more fluent BH4-dependent L-Trp hydroxylation reaction. In addition, the surplus of intermediate L-Trp was effectively relieved, which is more favorable for the practical application of this 5-HTP engineered strain.

**Table 1:** Sequences and relative strengths of M1 promoters

Promoters	Sequence ^a^	Strength ^b^
lacZ	**TTTACA**CTTTATGCTTCCGGCTCG**TATGTTG**TGTGGAATTGTGAGCGGATAACAATTTCACACAGGAAACAGCT	0.3
M1–12	TTATCTCTGGCGGTG**TTGACA**AGAGATAACAACGTTGA**TATAAT**TGAGCCCTTTTGGTGCGTCAGTCAGTTTAAACCAGGAAACAGCT	0.1 ± 0.01
M1–30	TTATCTCTGGCGGTG**TTGACA**AGAGATAACAACGTTGA**TATAAT**TGAGCCTGAGGTGGCTTATTATTCGTTTAAACCAGGAAACAGCT	0.7 ± 0.04
M1–46	TTATCTCTGGCGGTG**TTGACA**AGAGATAACAACGTTGA**TATAAT**TGAGCCTCTCGCCCCACCAATTCGGTTTAAACCAGGAAACAGCT	1.5 ± 0.1
M1–37	TTATCTCTGGCGGTG**TTGACA**AGAGATAACAACGTTGA**TATAAT**TGAGCCACTGGCTCGTAATTTATTGTTTAAACCAGGAAACAGCT	1.9 ± 0.12
M1–93	TTATCTCTGGCGGTG**TTGACA**AGAGATAACAACGTTGA**TATAAT**TGAGCCCGTATTGTTAGCATGTACGTTTAAACCAGGAAACAGCT	4.0 ± 0.12

^a^ Bold: −35 and − 10 regions of the promoter. Underlined: flanking sequence between promoters and RBS sequence

^b^ The promoter strength is represented as the relative strength in glucose media and aerobic conditions, where the strength of induced *lacZ* was promoted in LB medium and aerobic conditions

## Fed-batch fermentation of 5-HTP in a 5-L bioreactor

**Fig. 6:**
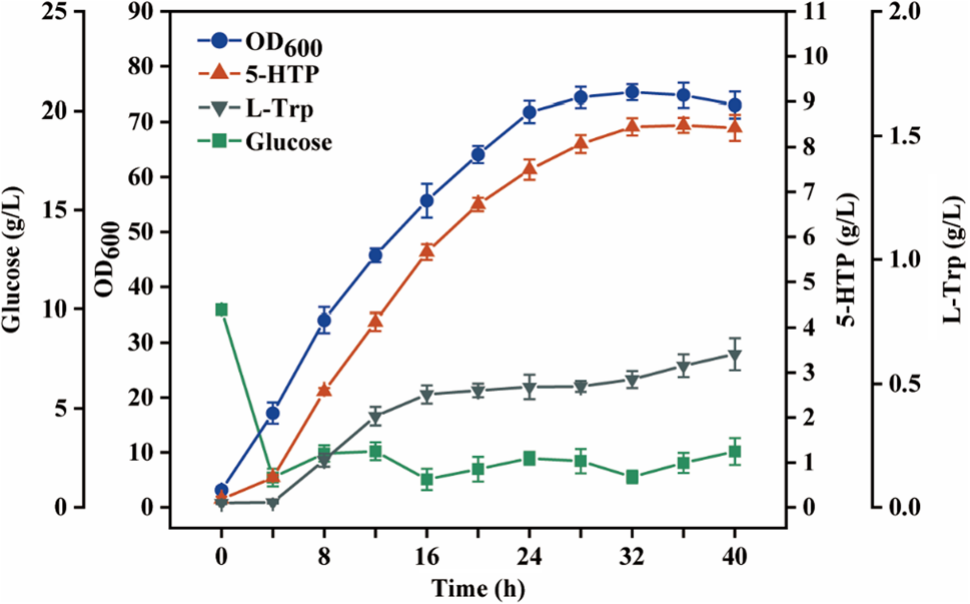
Fed-batch fermentation results of HTP11 strain in a 5-L bioreactor. The data are presented as averages, and the error bars represent standard deviations (n = 3 independent experiments)

To further evaluate the potential production performance of the engineered strain TPH11, fed-batch fermentation was conducted in a 5-L bioreactor. As shown in Fig. 6, the cells remained in logarithmic phase for the first 24 h of fermentation, and then gradually entered the stable phase, with the highest OD_600_ of 75.3 at 32 h. Meanwhile, the titer of 5-HTP increased exponentially after 4 h of fermentation and peaked at 32 h at 8.58 g/L; the average real-time yield and productivity both reached the maximum in the 4–8 h interval at 0.15 g/g glucose and 0.48 g/L/h, respectively (Fig. S7b and c), as well as the total yield up to 32 h of fermentation at 0.095 g/g glucose. To the best of our knowledge, this represents the highest titer and efficiency reported to date for fermentation of 5-HTP from glucose. Meanwhile, the synthesis of 5-HTP was found to be coupled with cell growth, which is consistent with the conclusions from previous studies [12]. In addition, measurable extracellular L-Trp concentrations were at extremely low levels (~ 0.5 g/L) for most of the fermentation, which is highly advantageous for downstream engineering.

## Discussion

With the growing market for 5-HTP, synthetic biologists are increasingly developing more efficient microbial cell factories to meet an increasing demand for 5-HTP. However, the large-scale microbial fermentation production of 5-HTP has yet to be achieved, the main reasons for this being the low activity and low solubility of hydroxylase in prokaryotic chassis cells [39, 40], the complexity of the synthesis and regeneration pathways of cofactors [41, 42], and the challenge of balancing the metabolic flow of the precursor L-Trp [12, 43]. These issues have resulted in an existing process that is currently ongoing in small laboratory trials and at the shake bottle scale, rendering it challenging to meet the volume requirements of practical applications. To overcome these bottlenecks, in this study, an efficient and controllable gene expression system was constructed, followed by the introduction of key heterologous pathways related to 5-HTP biosynthesis in engineered *E. coli*. Furthermore, these pathways were modularly reconstituted and systematically optimized to construct the L-Trp hydroxylation module, L-Trp biosynthesis module, and NAD(P)H regeneration module, resulting in a high-quality 5-HTP-producing strain. As a result, the highest production efficiency of this strain reported to date was achieved, showing great potential for use in industrial applications.

In the past decade, researchers have made extensive efforts to develop multiple pathway strategies (Table 2) by which to efficiently synthesize 5-HTP in *E. coli*. However, the BH4-dependent hydroxylation reaction pathway mediated by the truncated form of TPH2 is the most efficient and reliable. Despite this, the deficiency in hydroxylation activity of the mutant remains a key bottleneck limiting 5-HTP overproduction. Fortunately, with the development of protein engineering, adaptive laboratory evolution (ALE) has provided new technical support for the development of highly active hydroxylase mutants. Luo et al. [28] designed a genetic selection dependent on the tyrosine synthesis activity of phenylalanine hydroxylase and successfully acquired a protein mutant, TM1, with significantly increased protein abundance in *E. coli* using ALE. Subsequently, in a study on microbial melatonin production [21], the synthesis of 5-HTP was focused on as a rate-limiting step in its pathway. The method and results of Luo et al. were improved, resulting in a mutant TM2 with significantly increased tryptophan hydroxylation activity in *E. coli*. In this study, these new mutants were tested in *E. coli*, and TM2 stood out for its excellent hydroxylation activity and solubility, which was identified as the best choice for the heterologous synthesis of 5-HTP. In addition, although the synthesis and regeneration pathways of BH4 are well understood and have been applied in previous 5-HTP producing strains, the expression and regulation of related enzyme lines still need to be adjusted according to the actual application. Therefore, we designed two artificial mini-operons such that the enzymatic systems of BH4 synthesis and regeneration pathways could be co-expressed at different intensities. Moreover, the application of the “xylose-induced-T7RNAP gene expression system” enabled the efficient and stable expression of various heterologous genes. The *mlc* gene in this system was mutated to maintain glucose and xylose consumption at virtually identical rates [19]. These features facilitate the use of the second-generation feedstock xylose and allow the use of hemicellulose hydrolysate as a potential carbon source for 5-HTP fermentation.

It is well known that the endogenous L-Trp synthesis pathway in *E. coli* is characterized by a lengthy pathway and complex regulatory mechanisms, which make it difficult to modify engineered strains for the overproduction of L-Trp using synthetic biology [30, 44, 45]. The deregulation and overexpression of key enzymes, a common tool in metabolic engineering, can substantially enhance the smoothness of the target product synthesis pathway. Hence, in this study, efficient synthesis of 5-HTP was achieved by enrichment of the precursor L-Trp pool through modification of the *E. coli* native tryptophan operon and the overexpression of the feedback resistance genes t*rpE*^*fbr*^, *aroG*^*fbr*^, and *serA*^*fbr*^. Notably, *serA*^*fbr*^ overexpression contributed more significantly to the increase in 5-HTP, most likely because the NAD^+^-dependent rate-limiting reaction catalyzed by PGDH and the NADH-dependent BH4 regeneration pathway were mutually reinforced. Nevertheless, the intracellular reduced cofactor remained deficient (Fig. 5c) and the tryptophan surplus became more severe (Fig. 4a and Fig. S8).

The solution to the intermediate L-Trp surplus problem can be found in the following three ways: one is to weaken the tryptophan pathway to balance the L-Trp carbon flux and hydroxylation reaction efficiency; the second is to directly enhance the hydroxylation reaction by increasing the copy number of the hydroxylase expression plasmid; and the third is to indirectly enhance the hydroxylation reaction by promoting the regeneration of reduced cofactors. From previous experimental results (Fig. 4a), the weakening of the tryptophan pathway results in a huge reduction in 5-HTP production. More importantly, even though the tryptophan pathway was weak, L-Trp residues were still detected; therefore, the first scenario is not feasible with the pathway design of this study. In addition, the results of Xu’s study [46] showed that an increase in hydroxylase plasmid copy number would increase the metabolic burden of the cells and was not conducive to the over-synthesis of 5-HTP, which negates the second option. In contrast, the third option, that is, the construction of a reduced cofactor regeneration pathway, not only promotes the synthesis of BH4 and the regenerative pathway operation to enhance the intracellular L-Trp hydroxylation reaction indirectly, but also restores intracellular redox balance to maintain cell growth homeostasis, which seems to be a more secure and effective choice. Therefore, in this study, a GDH_*esi*_-mediated NAD(P)H regeneration module was constructed. Compared with other cofactor regeneration systems [37], this pathway not only reduces two reduced cofactors but also does not consume additional ATP, with economical and easy availability of the substrate glucose, which increases the cost-effectiveness of the whole process. Fortunately, the experimental results (Fig. 5) demonstrate that the strategy was effective. It was determined that a moderate intensity of NAD(P)H regeneration module could significantly reduce the surplus of intermediate L-Trp and better promote the recovery of cell growth, as well as the improve the 5-HTP titer and yield. It was also observed that an overpowered NAD(P)H regeneration pathway resulted in an imbalance in intracellular redox. Moreover, an overvalued NAD(P)H/NAD(P)^+^ ratio may reduce the flux of PRPP to L-Trp and negatively affect the NAD^+^-dependent PGDH reaction, both of which can cause a decrease in the precursor L-Trp and adversely affect 5-HTP production; this is because the conversion of NAD(P)H to NAD(P)^+^ is intertwined with multiple key metabolic pathways. These results emphasize the importance of reduced cofactors in 5-HTP production.

The results of the 5-L bioreactor fermentation demonstrated that the final strain HTP11 was more competitive in titer compared with the reported 5-HTP engineered strain (Table 2), which enabled the industrial production of 5-HTP. In addition, the residues of intermediate L-Trp at the end of fermentation are low, and therefore, the cost of purification will be reduced significantly, making it economically feasible for industrial applications. However, we also noted that 5-HTP production stalled after the cells entered the stable phase, suggesting that 5-HTP synthesis was dependent on cell growth. This conclusion is also supported by the fact that the yield and productivity showed a similar trend to that of the cell growth rate (Fig. S7). The optimization of a wide range of fermentation process conditions, including medium composition and culture parameters, may be the most direct and effective way to solve this problem.

**Table 2:** Production of 5-HTP by microorganisms

Host	Hydroxylases	Cofactors	Cultivation mode	medium type	Titer (g/L)	Reference
*E. coli*	Phenylalanine 4-hydroxylase form *Chromobacterium violaceum* with mutations of L101Y and W180F	BH4	Shake flask; Supplementation of 5 mM L-Trp	-	0.55	Hara et al., 2013 [11]
*E. coli*	Phenylalanine-4-hydroxylase from *Xanthomonas campestris* with a mutation of W179F	MH4	Shake flask; Supplementation of 2 g/L L-Trp	M9 minimal medium	1.1–1.2	Lin et al.,. 2014 [16]
*E. coli*	Aromatic amino acid hydroxylase from *Cupriavidus taiwanensis* with a mutation of W179F	BH4	Supplementation of 1 g/L L-Trp	Mineral medium	0.55	Mora-Villalobos et al., 2017 [44]
*E. coli*	Aromatic amino acid hydroxylase from *Cupriavidus taiwanensis* with mutations of F197 L and E219C	MH4	Fed-batch	Mineral medium	0.962	Mora-Villalobos et al., 2018 [17]
*E. coli*	Human TPH2 mutant with a deletion of first 145 N-terminal and 24 C-terminal aminoacids (TPH2, NΔ145/CΔ24)	BH4	Fed-batch; Glycerol as carbon source	Mineral medium	5.1	Wang et al., 2018 [12]
*E. coli*	Truncated human TPH2 (NΔ145/CΔ24)	BH4	Shake flask; Glycerol as carbon source	Mineral medium	1.61	Xu et al., 2020 [43]
*E. coli*	Truncated human TPH2 (NΔ145/CΔ24) with mutations of E2K, N97I and P99C	BH4	Fed-batch; Glucose as carbon source	Mineral medium	8.58	This study

## Materials and methods

### Chemicals and reagents

Unless otherwise stated, all chemicals and reagents were purchased from Shanghai Aladdin Bio-Chem Technology Co., Ltd. The reagent was, at the least, of analytical purity. Glucose monohydrate was purchased from Fufeng Biotechnology Co., Ltd. Ammonia solution was purchased from Sinopharm Chemical Reagent Co., Ltd. DNA polymerase was purchased from Dalian Bao Biological Co., Ltd. The oligonucleotide primers (Additional file 1: Table S1) were synthesized by Tianjin Jin Wei Zhi Biological Co., Ltd.

### Strains, plasmids, and culture conditions

The plasmids and the engineered strains used in this study are listed in Table 3. *E. coli* DH5α was used for plasmid vector construction and cloning, and *E. coli* K-12 w3110 was used as the starting strain for gene manipulation. Plasmids pRED-Cas9 and pGRB were used in CRISPR/Cas9-mediated gene editing [45]. The plasmid pSTV28 was used to express the tryptophan hydroxylase mutant.

Ampicillin (100 µg/mL) and spectinomycin (50 µg/mL) were used in the gene editing system when necessary. Isopropyl β-d-thiogalactoside (IPTG) and L-arabinose at final concentrations of 0.2 mM and 0.2% (w/v), respectively, were added to the induction experiments.

**Table 3:** Strains and plasmids used in this study

Strains	Characteristics	Source
* E. coli* DH5α	Host for cloning	Lab stock
* E. coli* K-12 w3110	Wild type, starting strain	Lab stock
HTP01	*E. coli* W3110, *∆tnaA*, *lacI*::P_*xylF*_-*T7RNAP*, *mlc*::mlc*	This study
HTP02	HTP01, *yghX*::P_*trc*_-*mtrA*-*PTPS*-*SPR*	This study
HTP03	HTP02, *yjgX*::P_*T7*_-*PCD*-*DHPR*	This study
HTP04	HTP03, pSTV-TM	This study
HTP05	HTP03, pSTV-TM1	This study
HTP06	HTP03, pSTV-TM2	This study
HTP07	HTP06, *trpLE*::P_*trc*_-*trpE*^*fbr*^	This study
HTP08	HTP07, *yjiV*::P_*trc*_-*aroG*^*fbr*^	This study
HTP09	HTP08, *ycgH*::P_*trc*_-*serA*^*fbr*^	This study
HTP10	HTP09, *mbhA*::P_*M1−12*_-*gdh*_*esi*_	This study
HTP11	HTP09, *mbhA*::P_*M1−30*_-*gdh*_*esi*_	This study
HTP12	HTP09, *mbhA*::P_*M1−46*_-*gdh*_*esi*_	This study
HTP13	HTP09, *mbhA*::P_*M1−37*_-*gdh*_*esi*_	This study
HTP14	HTP09, *mbhA*::P_*M1−93*_-*gdh*_*esi*_	This study
Plasmid		
pGRB	gRNA expression vector	Lab stock [45]
pRed-cas9	Cas9 expression vector	Lab stock [45]
pSTV28	P15A ori, CmR, *E.coli* expression vector	Lab stock
pSTV-TM	pSTV28, P_*T7*_-*TM*	This study
pSTV-TM1	pSTV28, P_*T7*_-*TM1* (*TM*_*E2K*_)	This study
pSTV-TM2	pSTV28, P_*T7*_-*TM2* (*TM*_*E2K/N97I/P99C*_)	This study

### Acquisition of target genes

The heterologous target genes used in this study were artificially synthesized by codon optimization (Jin Weiji, Tianjin, China). The endogenous target genes were amplified using *E. coli* K-12 w3110 genomic DNA as a template.

*TM2* gene preparation was used to describe the process of point mutation gene construction. First, the *TM* gene was used as a template to obtain upstream and downstream mutant gene fragments using primer pairs TM2-1/TM2-2 and TM2-3/TM2-4, respectively, where TM2-1 contained mutated bases G4A and G6A, and TM2-2 and TM2-3 contained both mutated bases A290T, T291C, C295T, C296G, and T297C, and complementary bases in the reverse direction. The two gene fragments were then purified and mixed, and the *TM2* gene was obtained by overlapping PCR using primer pairs TM2-1/TM2-4.

### Recombinant plasmid construction

Recombinant plasmids were constructed by homologous recombination (Fig. S3). The construction of *TM* gene overexpression plasmid pSTV-TM is an example. First, primers P-TM-S, P-TM-A, P-line-S, and P-line-A were designed using CE Design V1.03, each with the corresponding homologous sequence. Subsequently, the target fragment and linear vector were amplified using the primer pairs P-TM-S/P-TM-A and P-line-S/P-line-A, respectively. Finally, the linear vector was ligated to the target gene fragment by homologous recombination using a ClonExpress II one-step cloning kit (Vazyme Biotech, Nanjing, China).

### Genome editing

The modification of the *E. coli* K-12 w3110 genome was accomplished using a CRISPR/Cas9-mediated gene editing system [46]. The deletion of the *tnaA* gene was used to describe the genomic manipulation process. First, a 20-bp spacer region sequence was obtained using CRISPR RGEN Tools (http://www.rgenome.net/). Then, a pair of complementary primers (pGRB-tnaA-1 and pGRB-tnaA-2) was synthesized and annealed to form a dsDNA segment containing the spacer region sequence and flanking sequences homologous to the pGRB backbone. The dsDNA was subsequently ligated to the linearized pGRB plasmid using the ClonExpress® II Seamless Cloning Kit (Vazyme, China) to construct the pGRB-tnaA plasmid. In addition, the upstream and downstream homologous fragments of *tnaA* were amplified with the primers tnaA-1/tnaA-2 and tnaA-3/tnaA-4, respectively, and ligated by overlapping polymerase chain reaction to obtain the donor DNA fragment (DNA-tnaA). Next, DNA-tnaA and pGRB-tnaA were co-transformed into electrocompetent *E. coli* W3110 cells harboring pRED-Cas9. The cells were cultured in 1 mL of SOC media at 32 °C for 2 h, inoculated in LB plates containing spectinomycin and ampicillin, and incubated at 37 °C for 15 h. Positive transformants were screened by colony polymerase chain reaction and confirmed by sequencing (Golden Vizi). In addition, 0.2% L-arabinose could be used to cure the pGRB-tnaA plasmid of the correct colonies. When the above molecular manipulation was complete, the culture temperature was increased from 37 to 42 °C to eliminate the plasmid pRED-Cas9. When the target gene is integrated into the chromosome, a donor DNA fragment is obtained by ligating the two homologous arms and integrating the gene. The other steps were the same as those previously described.

### Cultivation in shake flasks

The engineered bacteria cultured on agar slants were transferred to a 500-mL covered baffled shake flask containing 30 mL of seed medium and incubated at a shaker temperature of 36 °C and a speed of 200 rpm for 10 h. The seed medium contained (per liter) 20 g glucose, 5 g yeast extract, 2 g peptone, 2 g KH_2_PO_4_, 4 g (NH_4_)_2_SO_4_, 1 g MgSO_4_·7H_2_O, 2 mg V_B1_, 2 mg V_B3_, 2 mg V_B5_, 2 mg V_B12_, 2 mg V_H_, 30 mg chloramphenicol, and 8 mg phenol red at pH 7.0–7.2. The seed cultures (3 mL) were inoculated in 500-mL shake flasks containing 30 mL of fermentation medium and incubated for 26 h at 36 °C with a shaker speed of 200 rpm. The fermentation medium contained (per liter) 20 g glucose, 4 g citric acid monohydrate, 5 g yeast extract, 2 g yeast extract, 2 g peptone, 6 g KH_2_PO_4_, 4 g (NH_4_)_2_SO_4_, 2 g MgSO_4_·7H_2_O, 10 mg MnSO_4_·H_2_O, 50 mg FeSO_4_·7H_2_O, 2 mg V_B1_, 2 mg V_B3_, 2 mg V_B5_, 2 mg V_B12_, 2 mg V_H_, 30 mg chloramphenicol, and 8 mg phenol red at pH 7.0–7.2. The pH of the fermentation process was maintained using ammonium hydroxide (25%, v/v) throughout the fermentation process, depending on the color change of phenol red. A sterile glucose solution (60% w/v) was supplied when glucose was depleted in the initial culture broth to meet the carbon source requirements for cell growth and product synthesis. A single addition of xylose 5 g/L at the beginning of fermentation induced gene expression, driven by the T7 promoter.

### Fed-batch fermentation in a 5-L bioreactor

The appropriate amount of agar slant culture cells was transferred to 3 L of seed medium in a 5-L bioreactor (Baoxing, Shanghai, China). The seed and fermentation media in the bioreactor were the same as those used in the shake flasks, except that phenol red was not added. When the OD_600_ reached 12–15, 600 mL of seed culture was retained and fresh fermentation medium was added immediately to make a final volume of fermentation broth of 3 L. The pH was automatically controlled throughout the fermentation process by adding ammonium hydroxide (25%, v/v) at 36 °C, and the dissolved oxygen was maintained above 25% by varying the stirring rate and aeration. Gene expression driven by the T7 promoter was induced by the addition of xylose at a final concentration of 5 g/L at the beginning of fermentation. When the substrate glucose was depleted, sterile glucose solution (80%, w/v) was supplemented in appropriate amounts, and the glucose concentration was maintained below 5 g/L.

### Analytical methods

The absorbance at 600 nm was measured using a UV/VIS spectrophotometer (UV1800; Shanghai Essence Technology Instruments Co., Ltd., Shanghai, China) to determine cell growth status. A biosensor analyzer (SBA-40E; Shandong Academy of Sciences, Shandong, China) was used to determine glucose concentration. The NAD(P)H and NAD(P)^+^ levels of the cells were detected using NADPH/NADP^+^ and NADH/NAD^+^ analysis kits with WST-8 (S0179 and S0175; Beyotime, China). The concentrations of L-Trp and 5-HTP standards and samples were detected by high-performance liquid chromatography (Thermo U-3000 series with UV absorption detector) using an Agilent Reverse TC-C18(2) column at 25 °C. The mobile phase was a mixture of methanol and 10 mM potassium phosphate buffer (pH 6.5) (12:88, v/v) at a flow rate of 1 mL/min.

### Statistical analysis

Data represent the mean and standard deviation (SD) of three independent experiments. One-way analysis of variance (ANOVA) and Dunnett’s multiple comparison test were used to determine significant differences between the data. 0.01 < P < 0.05 was considered significant, while P < 0.01 was considered highly significant.

## Supplementary Information

**Additional file 1: Tab. S1.** Primers used for strain construction in this study. **Tab. S2.** The synthesized heterologous protein sequence applied in this study.** Fig. S1.** Partial human physiological activities in which 5-HTP is involved and applications of 5-HTP in medication and health care.** Fig. S2.** Different pathways of 5-HTP heterologous synthesis.** Fig. S3. **Construction of tryptophan hydroxylase mutant gene expression plasmids pSTV-TM, pSTV-TM1 and pSTV-TM2.** Fig. S4.** 5-HTP titer per unit cell mass of HTP04-06 strains.** Fig. S5.** Intracellular NAD(P)H level of HTP09 and HTP11 strains.** Fig. S6.** HPLC detection information of 5-HTP and L-Trp.** Fig. S7.** Real-time efficiency of 5-HTP production by HTP11 in a 5-L bioreactor.** Fig. S8.** The ratio of tryptophan to 5-HTP in shake flask fermentation results of HTP07-09 and 10 strains. **Fig. S9.** Glucose consumption of HTP11 strain in a 5-L bioreactor.

## Data Availability

All data generated or analyzed during this study are included in this article and its Additional file.

## Abbreviations

G6P: Glucose-6-phosphate
F6P: Fructose-6-phosphate
GAP: Glyceraldehyde-3-phosphate
G3P: Glycerate-3-phosphate
PEP: Phosphoenolpyruvate
DAHP: 3 Deoxy-α-D-arabinoheptulosonate-7-phosphate
CHO: Chorismate
Gln: Glutamine
ANT: Anthranilate
3PHP: 3-Phosphohydroxypyruvate
3PS: 3-Phosphoserine
IND: Indole
Ribu5P: 5-Ribulose-5-phosphate
Xyl5P: Xylulose-5-phosphate
Rib5P: Ribose-5-phosphate
Sed7P: Sedoheptulose-7-phosphate
E4P: Erythrose 4-phosphate
PRPP: Phosphate-5-riobosylpyrophosphate
IMP: Inosinate
GMP: Guanosine monophosphate
GTP: Guanosine triphosphate
NH2TP: 7,8-Dihydroneopterin 3-triphosphate
6PPH4: 6-Pyruvoyltetrahydropterin
BH4: Tetrahydrobiopterin
BH3OH: Pterin-4α-carbinolamine
5-HIND: 5-Hydroxyindole
*esi*: *Exiguobacterdium sibiricum*
*bus*: *Bacillus subtilis*
*has*: *Human*
