# Supplementary material for: Metabolic engineering of Escherichia coli for efficient production of L-5-hydroxytryptophan from glucose
Source: Microb Cell Fact. 2022 Sep 24;21:198. doi: 10.1186/s12934-022-01920-3 (PMC9509612; doi:10.1186/s12934-022-01920-3)
Supplement: Supplementary file 1 — Additional file 1: Tab. S1. Primers used for strain construction in this study. Tab. S2. The synthesized heterologous protein sequence applied in this study. Fig. S1. Partial human physiological activities in which 5-HTP is involved and applications of 5-HTP in medication and health care. Fig. S2. Different pathways of 5-HTP heterologous synthesis. Fig. S3. Construction of tryptophan hydroxylase mutant gene expression plasmids pSTV-TM, pSTV-TM1 and pSTV-TM2. Fig. S4. 5-HTP titer per unit cell mass of HTP04-06 strains. Fig. S5. Intracellular NAD(P)H level of HTP09 and HTP11 strains. Fig. S6. HPLC detection information of 5-HTP and L-Trp. Fig. S7. Real-time efficiency of 5-HTP production by HTP11 in a 5-L bioreactor. Fig. S8. The ratio of tryptophan to 5-HTP in shake flask fermentation results of HTP07-09 and 10 strains. Fig. S9. Glucose consumption of HTP11 strain in a 5-L bioreactor. [file 12934_2022_1920_MOESM1_ESM.docx]

**Supplementary information**

# Metabolic engineering of *Escherichia coli* for efficient production of L-5-hydroxytryptophan from glucose

Zhen Zhang^1,3^, Zichen Yu^1,3^, Jinduo Wang^1,3^, Yifa Yu^2^, Pengjie Sun^1,3^, Xiaoguang Fan^1,3^, Qingyang Xu^1,3*^

1 *College of Biotechnology, Tianjin University of Science & Technology, Tianjin, 300457, P. R. China.*

*2 Nanning Harworld Biological Technology Co., Ltd, Nanning, 530000, P. R. China.*

3 *Key Laboratory of Industrial Fermentation Microbiology, Ministry of Education, Tianjin University of Science & Technology, Tianjin, 300457,* *P. R. China.*

**Tab. S1** Primers used for strain construction in this study.

**Tab. S2** The synthesized heterologous protein sequence applied in this study.

**Fig.S1** Partial human physiological activities in which 5-HTP is involved and applications of 5-HTP in medication and health care.

**Fig.S2** Different pathways of 5-HTP heterologous synthesis.

**Fig.S3** Construction of tryptophan hydroxylase mutant gene expression plasmids pSTV-TM, pSTV-TM1 and pSTV-TM2.

**Fig.S4** 5-HTP titer per unit cell mass of HTP04-06 strains.

**Fig.S5** Intracellular NAD(P)H level of HTP09 and HTP11 strains.

**Fig.S6** HPLC detection information of 5-HTP and L-Trp.

**Fig.S7** Real-time efficiency of 5-HTP production by HTP11 in a 5-L bioreactor.

**Fig.S8** The ratio of tryptophan to 5-HTP in shake flask fermentation results of HTP07-09 and 10 strains.

**Fig.S9** Glucose consumption of HTP11 strain in a 5-L bioreactor.

Tab. S1 Primers used for strain construction

| Primers | Sequence (5’→ 3’) |
| --- | --- |
| TM-1 | ATGGAGGAAGAGCTGGAAGATG |
| TM-2 | TTATGTATCTTTCAAAATTTCGATACTCTG |
| TM2-1 | ATGAAACTGGAAGATGTACCGTGGTTCCCG |
| TM2-2 | TTTGGTCAGTAAGCAAAAGATCTTCAGATATTC |
| TM2-3 | GAATATCTGAAGATCTTTTGCTTACTGACCAAA |
| TM2-4 | TTATGTATCTTTCAAAATTTCGATACTCTG |
| P-line-S | ATCCCAATGGCGCGCCGA |
| P-line-A | ATCCCAATACGCGTCAATTCA |
| P-TM-S | GAATTGACGCGTATTGGGATTAATACGACTCACTATAGGGGAATTGTG |
| P-TM-A | GCTCGGCGCGCCATTGGGATCAAAAAACCCCTCAAGACCCG |
| tnaA-1 | ATTGATGGTCTTGAACAATTGGC |
| tnaA-2 | GAAGCTGTCGTCTTTCATGCACATTTTACTGGCTCAATAACACGAATG |
| tnaA-3 | CATTCGTGTTATTGAGCCAGTAAAATGTGCATGAAAGACGACAGCTTC |
| tnaA-4 | TCATGATGCCACCTTTAGAGGAA |
| lacIZ-1 | ACAACAACTGGCGGGCAAAC |
| lacIZ-2 | TTGTGAATTATCTCCGCCGAGACAGAACTTAATGGG |
| lacIZ-3 | ATACTGTGCCGGGGCGAGTTGCGTGACTACCT |
| lacIZ-4 | CACAGCGGATGGTTCGGATA |
| PxylF-1 | GTTCTGTCTCGGCGGAGATAATTCACAAGTGTGCGCT |
| PxylF-2 | TTAATCGTGTTCATTTAGTGCCTCTTCCAGTTAGTAAATCC |
| T7RNAP-1 | ACTGGAAGAGGCACTAAATGAACACGATTAACATCGCTAAGA |
| T7RNAP-2 | CGCAACTCGCCCCGGCACAGTATCAAGGTATTT |
| mlc-1 | TTGAAGTGCTTTACCATAGCCTACA |
| mlc-3 | AAGGCGCTTCAATCATGAACATAACTCAATTTGTAGGGTCATAGTAATCCAGCAACTTCACGGCTGCCTCGAAAC |
| mlc-4 | TACTATGACCCTACAAATTGAGTTATGTTCATGATTGAAGCGCCTTTACTACGAAATAATACGATCCAGCAATGG |
| mlc-5 | TTAAAAAATGTTAACCCTGCAACAG |
| mlc*-1 | TTGAAGTGCTTTACCATAGCCTACA |
| mlc*-2 | TTAAAAAATGTTAACCCTGCAACAG |
| yjgX-1 | GGAAGTCAACGGGTTATGCG |
| yjgX-2 | TATTTCTAGAGGGGAATTGTTATCCGCTCACAATTCCCCTATAGTGAGTCGTATTAAAAATCACCACGAATACCAGAATC |
| yjgX-3 | GCGGATAACAATTCCCCTCTAGAAATAATTTTGTTTAACTTTAAGAAGGAGATATACCATGGCTGGTAAAGCTCACCGTC |
| yjgX-4 | CAAGACCCGTTTAGAGGCCCCAAGGGGTTATGCTAGTTAGAAGTAAGCCGGGGTCAGTTC |
| trpLE-1 | TTCTGTCTGCTGCGCGAGGAACT |
| trpLE-2 | CCTAGAAGAAATCAACCAGCGCATCAGAAAGTCTCCTGTGCATTGTCGATACCCTTTTTACGTGAACT |
| trpLE-3 | TGCGCTGGTTGATTTCTTCTAGGGTCATAGTAATCCAGCAACTATGGCTGACATTCTGCTGCTCGATA |
| trpLE-4 | GTTCCAGCAGGCGAGCGCCCTG |
| trpE^fbr^-2 | TACGAGCCGGATGATTAATTGTCAATGTCGATACCCTTTTTACGTGAACT |
| trpE^fbr^-3 | AGTTCACGTAAAAAGGGTATCGACATTGACAATTAATCATCCGGCTCGTA |
| trpE^fbr^-4 | TATCGAGCAGCAGAATGTCAGCCATTCAGAAAGTCTCCTGTGCATGATGC |
| trpE^fbr^-5 | GCATCATGCACAGGAGACTTTCTGAATGGCTGACATTCTGCTGCTCGATA |
| yghX-1 | GGTAGGCGGGATGACGGCATT |
| yghX-2 | GAAATTGTTATCCGCTCACAATTCCACACATTATACGAGCCGGATGATTAATTGTCAACGCTTGCGGATAGCGTTGCA |
| yghX-3 | CTCGTATAATGTGTGGAATTGTGAGCGGATAACAATTTCACACAGGAAACAGACCATGAAAGAAGTTAATAAAGAGCAAATCGAAC |
| yghX-4 | AAACAACAGATAAAACGAAAGGCCCAGTCTTTCGACTGAGCCTTTCGTTTTATTTGTTAGATGTCGTAGAAGTCAACGTGAGCAC |
| yjiV-1 | TAAAAGCACTACCTGTGAAGGGATG |
| yjiV-2 | AATTGTTATCCGCTCACAATTCCACACATTATACGAGCCGGATGATTAATTGTCAAACAGAATGCAACTTCGTATACAGGG |
| yjiV-3 | CTCGTATAATGTGTGGAATTGTGAGCGGATAACAATTTCACACAAGGAGATATACCATGAATTATCAGAACGACGATTTACGCAT |
| yjiV-4 | AACAACAGATAAAACGAAAGGCCCAGTCTTTCGACTGAGCCTTTCGTTTTATTTGTTACCCGCGACGCGCTTTTAC |
| ycgh-1 | TAAACTCGTCAGCGGCACAA |
| ycgh-2 | AATTGTTATCCGCTCACAATTCCACACATTATACGAGCCGGATGATTAATTGTCAAGGTAGGCGTTTCTGTTGATTCTG |
| yghX-3 | TTGTGAGCGGATAACAATTTCACACAAGGAGATATACCATGGCAAAGGTATCGCTGG |
| ycgH-4 | GACAAACAACAGATAAAACGAAAGGCCCAGTCTTTCGACTGAGCCTTTCGTTTTATTTGTTAGTACAGCAGACGGGCGCG |
| mbhA-1 | GCCAGCACGAACATAATCCC |
| mbhA-4 | CACCGACAAACAACAGATAAAACGAAAGGCCCAGTCTTTCGACTGAGCCTTTCGTTTTATTTGTTAACCGCGGCCCGC |
| mbhA-5 | AAAGACTGGGCCTTTCGTTTTATCTGTTGTTTGTCGGTGAACGCTCTCCTGAGTAGGACAAATGACCAAAAGTGCGTCCGATAC |
| mbhA-6 | CGGCGTAATCACAAACTGGC |
| mbhA-M112-2 | ACGCACCAAAAGGGCTCAATTATATCAACGTTGTTATCTCTTGTCAACACCGCCAGAGATAACACGGTGGCAGGTTTTGG |
| mbhA-M112-3 | ACGTTGATATAATTGAGCCCTTTTGGTGCGTCAGTCAGTTTAAACCAGGAAACAGCTATGGGCTATAACAGCCTGAAAGG |
| mbhA-M130-2 | TAAGCCACCTCAGGCTCAATTATATCAACGTTGTTATCTCTTGTCAACACCGCCAGAGATAACACGGTGGCAGGTTTTGG |
| mbhA-M130-3 | ACGTTGATATAATTGAGCCTGAGGTGGCTTATTATTCGTTTAAACCAGGAAACAGCTATGGGCTATAACAGCCTGAAAGG |
| mbhA-M137-2 | TTACGAGCCAGTGGCTCAATTATATCAACGTTGTTATCTCTTGTCAACACCGCCAGAGATAACACGGTGGCAGGTTTTGG |
| mbhA-M137-3 | ACGTTGATATAATTGAGCCACTGGCTCGTAATTTATTGTTTAAACCAGGAAACAGCTATGGGCTATAACAGCCTGAAAGG |
| mbhA-M146-2 | GGTGGGGCGAGAGGCTCAATTATATCAACGTTGTTATCTCTTGTCAACACCGCCAGAGATAACACGGTGGCAGGTTTTGG |
| mbhA-M146-3 | ACGTTGATATAATTGAGCCTCTCGCCCCACCAATTCGGTTTAAACCAGGAAACAGCTATGGGCTATAACAGCCTGAAAGG |
| mbhA-M193-2 | GCTAACAATACGGGCTCAATTATATCAACGTTGTTATCTCTTGTCAACACCGCCAGAGATAACACGGTGGCAGGTTTTGG |
| mbhA-M193-3 | ACGTTGATATAATTGAGCCCGTATTGTTAGCATGTACGTTTAAACCAGGAAACAGCTATGGGCTATAACAGCCTGAAAGG |
| pGRB-mlc-1 | GTCCTAGGTATAATACTAGT***TTCAGCATGATATCAGCGCA***GTTTTAGAGCTAGAA |
| pGRB-mlc-2 | TTCTAGCTCTAAAAC***TGCGCTGATATCATGCT***GAAACTAGTATTATACCTAGGAC |
| pGRB-mlc*-1 | AGTCCTAGGTATAATACTAGT***ATGAACATAACTCAATTTGT***GTTTTAGAGCTAGAA |
| pGRB-mlc*-2 | TTCTAGCTCTAAAAC***ACAAATTGAGTTATGTTCAT***ACTAGTATTATACCTAGGACT |
| pGRB-tnaA-1 | AGTCCTAGGTATAATACTAGT***CACTCGCGCTTATCGTGAAG***GTTTTAGAGCTAGAA |
| pGRB-tnaA-2 | TTCTAGCTCTAAAAC***CTTCACGATAAGCGCGAGTG***ACTAGTATTATACCTAGGACT |
| pGRB-yghX-1 | AGTCCTAGGTATAATACTAGT***TTTTATGGTCGTCAGGCACC***GTTTTAGAGCTAGAA |
| pGRB-yghX-2 | TTCTAGCTCTAAAAC***GGTGCCTGACGACCATAAAA***ACTAGTATTATACCTAGGACT |
| pGRB-yjgX-1 | AGTCCTAGGTATAATACTAGT***CCGTGGCTGAATACGGCACC***GTTTTAGAGCTAGAA |
| pGRB-yjgX-2 | TTCTAGCTCTAAAAC***CCGGGTGCCGTATTCAGCCA***ACTAGTATTATACCTAGGACT |
| pGRB-trpE-1 | AGTCCTAGGTATAATACTAGT***CTCGAACTGCTAACCTGCGA***GTTTTAGAGCTAGAA |
| pGRB-trpE-2 | TTCTAGCTCTAAAAC***TCGCAGGTTAGCAGTTCGAG***ACTAGTATTATACCTAGGACT |
| pGRB-trpE^bfr^-1 | AGTCCTAGGTATAATACTAGT***TGCGCTGGTTGATTTCTTCT***GTTTTAGAGCTAGAA |
| pGRB-trpE^bfr^-2 | TTCTAGCTCTAAAAC***AGAAGAAATCAACCAGCGCA***ACTAGTATTATACCTAGGAT |
| pGRB-yjiV-1 | AGTCCTAGGTATAATACTAGT***GCGTAGTCGAAATTCTCAGC***GTTTTAGAGCTAGAA |
| pGRB-yjiV-2 | TTCTAGCTCTAAAAC***GCTGAGAATTTCGACTACGC***ACTAGTATTATACCTAGGACT |
| pGRB-ycgH-1 | AGTCCTAGGTATAATACTAGT***ATGCGTCTGAACGACCGTGC***GTTTTAGAGCTAGAA |
| pGRB-ycgH-2 | TTCTAGCTCTAAAAC***GCACGGTCGTTCAGACGCAT***ACTAGTATTATACCTAGGACT |
| pGRB-mbhA-1 | AGTCCTAGGTATAATACTAGT***GCGTGATGTGAATGAGAAAA***GTTTTAGAGCTAGAA |
| pGRB-mbhA-2 | TTCTAGCTCTAAAAC***TTTTCTCATTCACATCACGC***ACTAGTATTATACCTAGGACT |


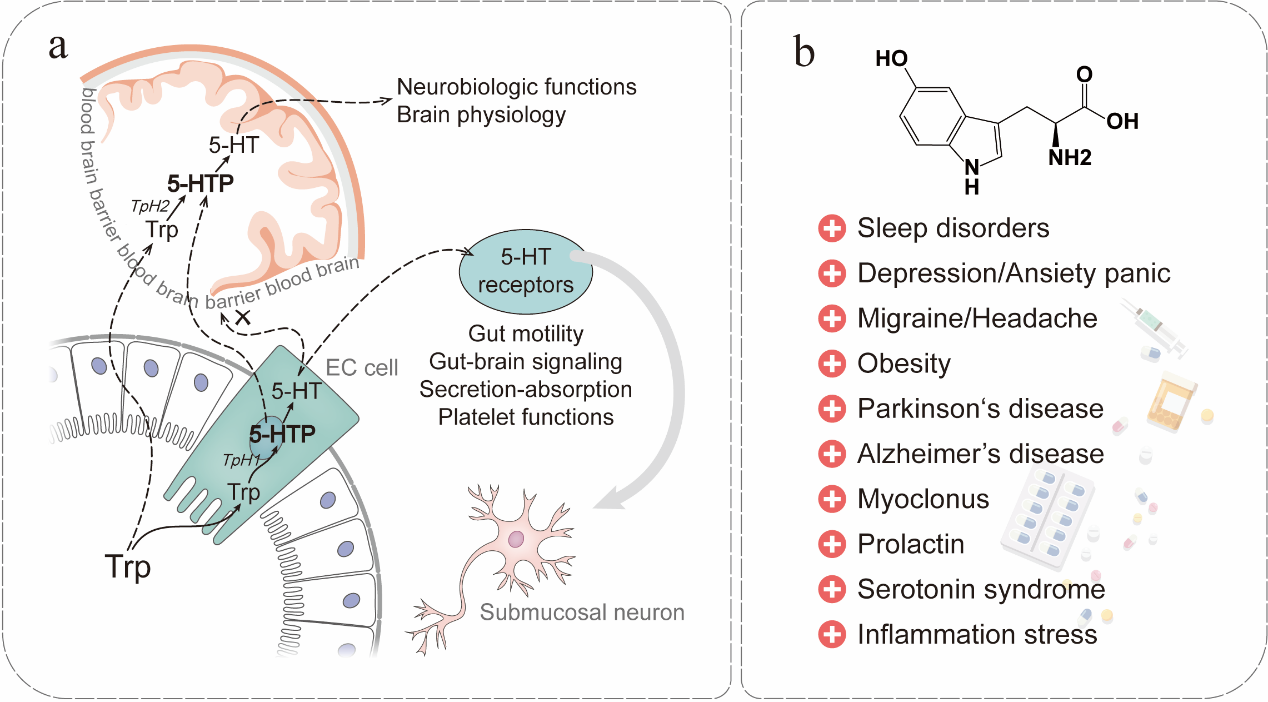


Fig. S1 (a) Partial human physiological activities in which 5-HTP is involved [1]. As a human neurotransmitter, 5-HT is mainly secreted in the brain and gut (enterochromaffin and CE cells), and is involved in a wide range of human physiological processes, including the transmission of gut-brain signaling, intestinal peristalsis and motility, secretion, vasodilatation, and nutrient absorption. Whereas 5-HTP, the direct precursor of 5-HT, is synthesized by the essential amino acid L-tryptophan via tryptophan hydroxylase Ⅱ (TPH2, in human brain) and tryptophan hydroxylase Ⅰ (TPH1, in EC cells), respectively; these reactions are also rate-limiting steps in 5-HT biosynthesis. (b) Applications of 5-HTP in medication and health care [2].


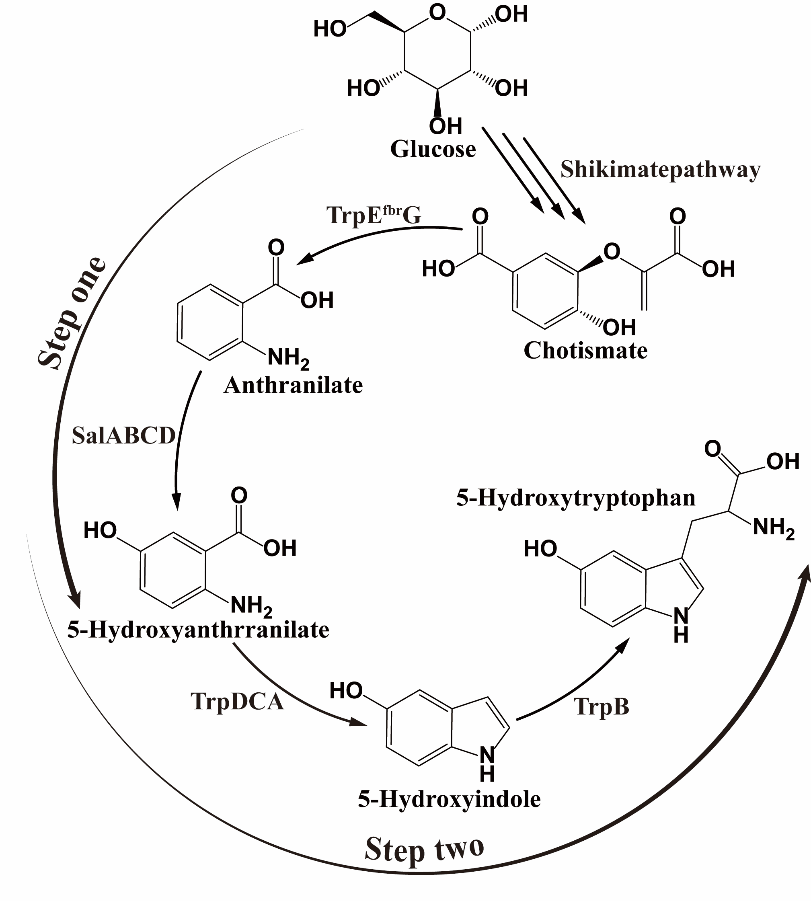


(a)


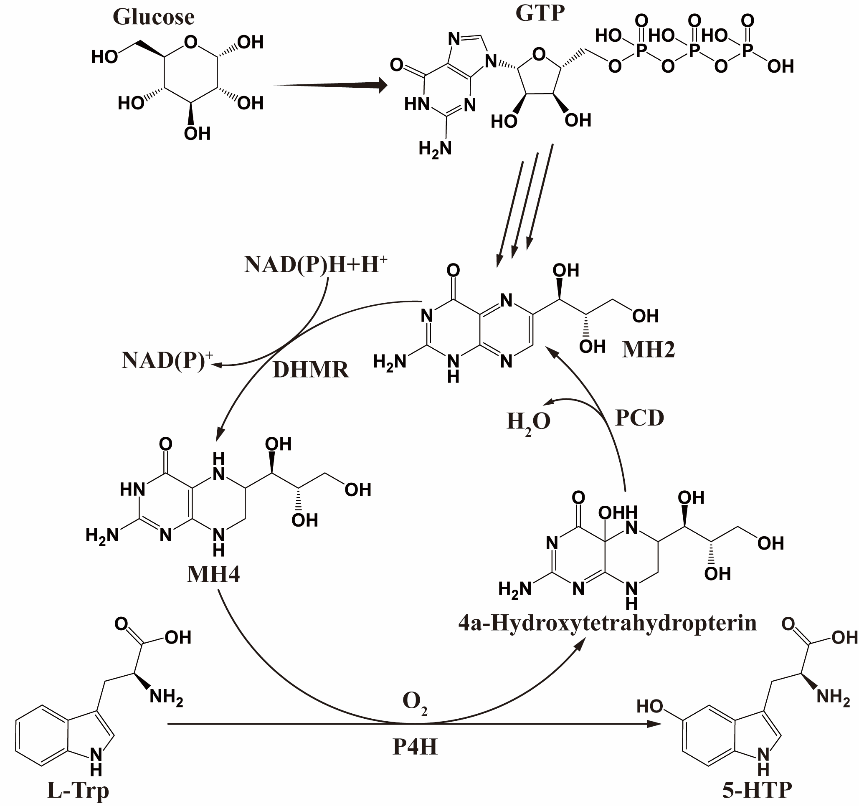


(b)


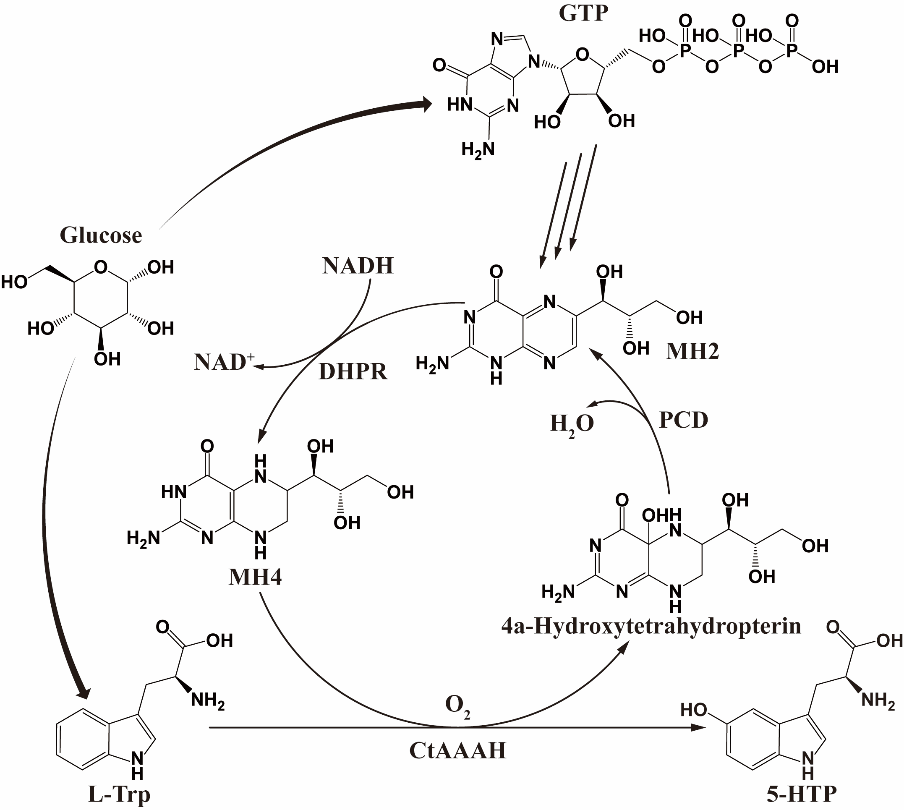


(c)


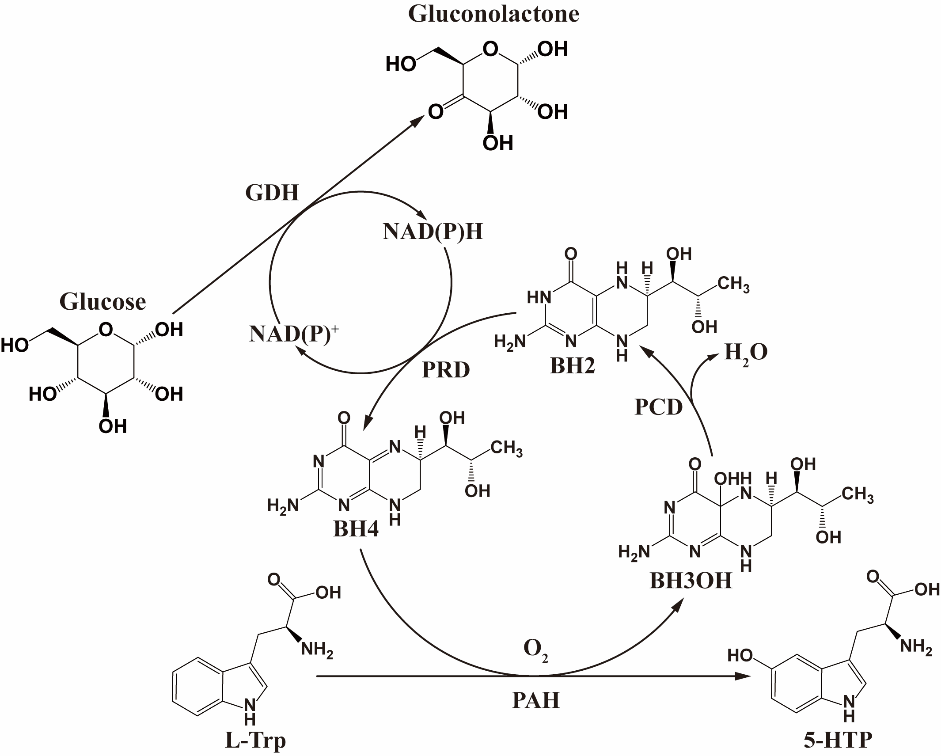


(d)


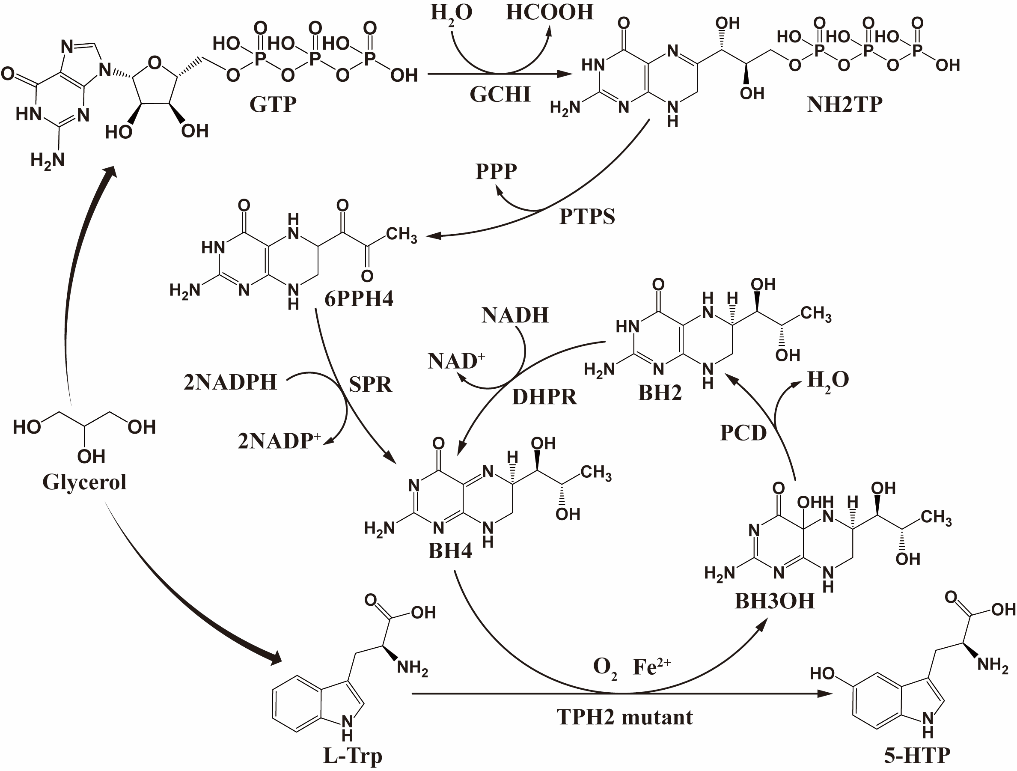


(e)

Fig S2. Different pathways of 5-HTP heterologous synthesis. (a) Strategy for synthesis 5-HTP in two steps [3]. (b) Phenylalanine 4‑hydroxylase (P4H)-mediated tryptophan hydroxylation pathway [4]. (c) Aromatic amino acid hydroxylase (CtAAAH)-mediated tryptophan hydroxylation pathway [5]. (d) Enhanced synthesis of 5-HTP through tetrahydropterin regeneration [6]. (e) Human TPH2 mutant-mediated tryptophan hydroxylation pathway [7].


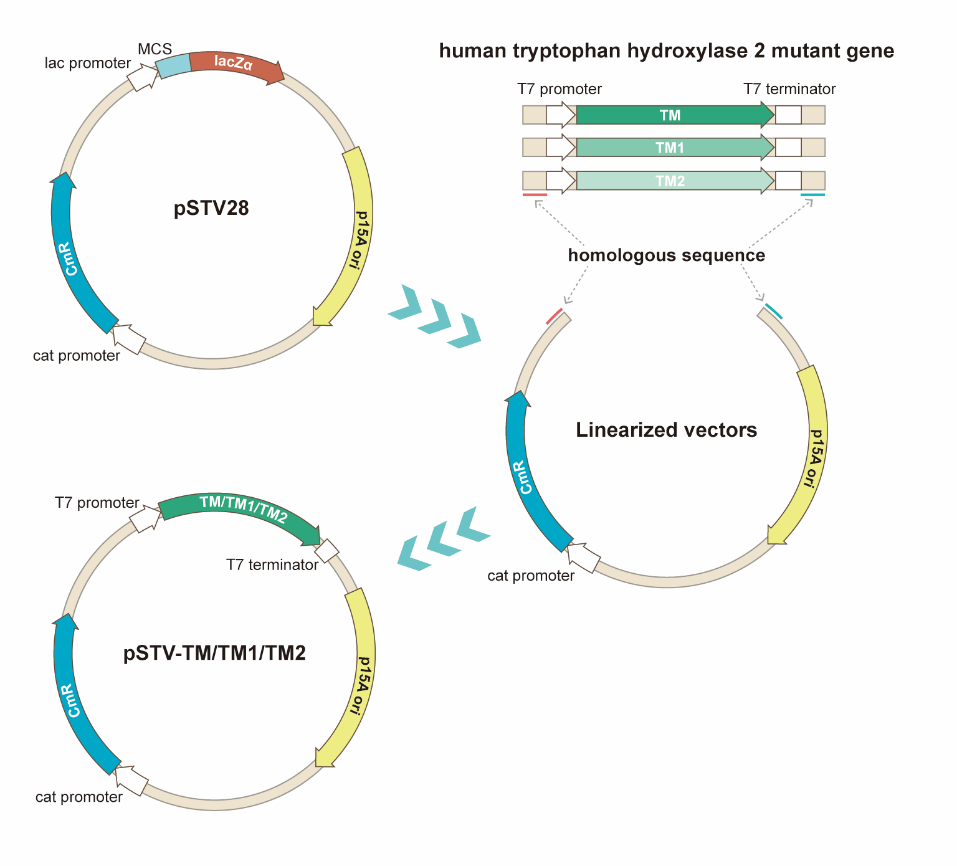


Fig. S3 Construction of tryptophan hydroxylase mutant gene expression plasmids pSTV-TM, pSTV-TM1 and pSTV-TM2.


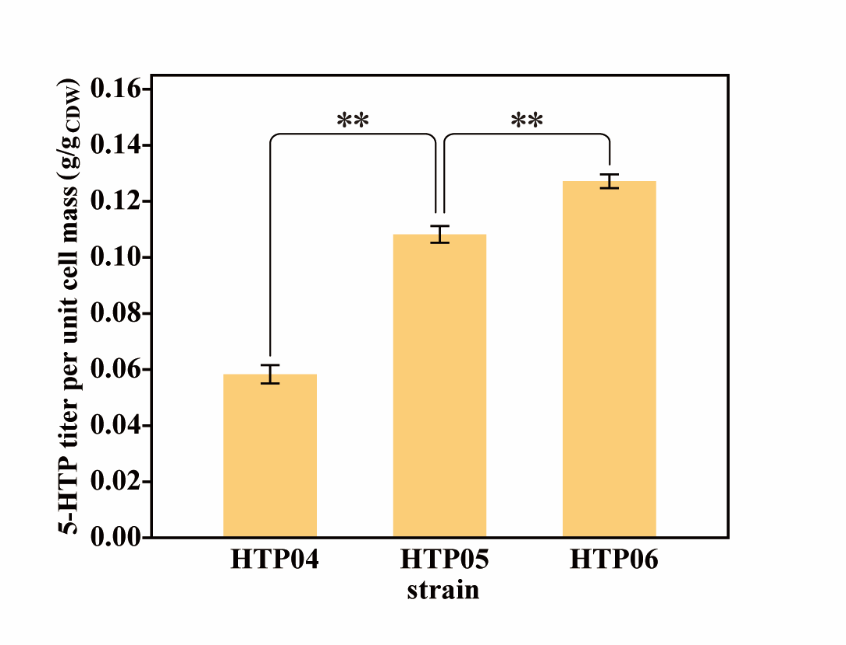


Fig. S4 5-HTP titer per unit cell mass of HTP04-06 strains.

The correlation relating the dry cell weight to the OD_600_ for our 5-HTP production strains was determined as 0.351 ± 0.011 g/L per OD_600_. The data are presented as averages, and the error bars represent standard deviations (n = 3 independent experiments). ** P < 0.01.


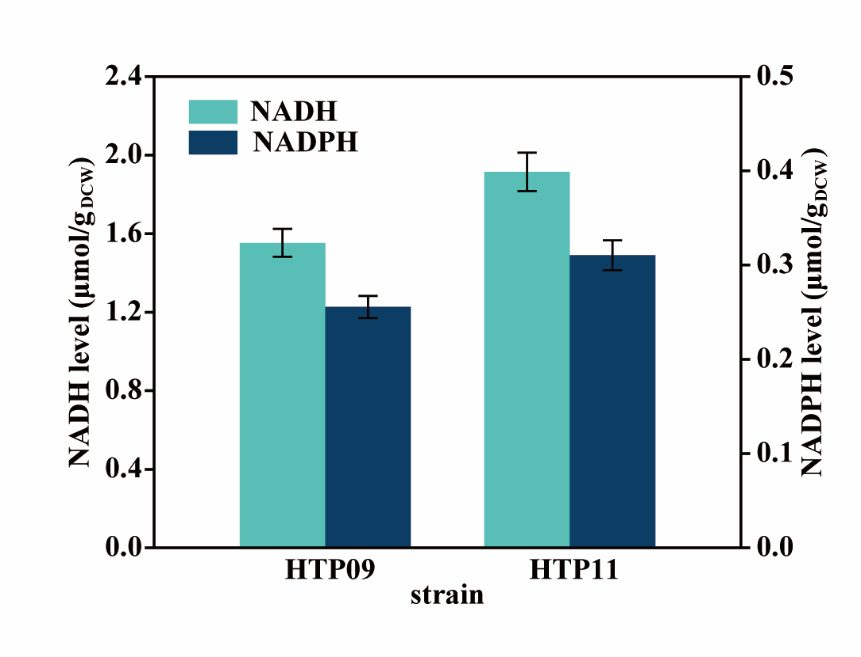


Fig. S5 Intracellular NAD(P)H level of HTP09 and HTP11 strains.


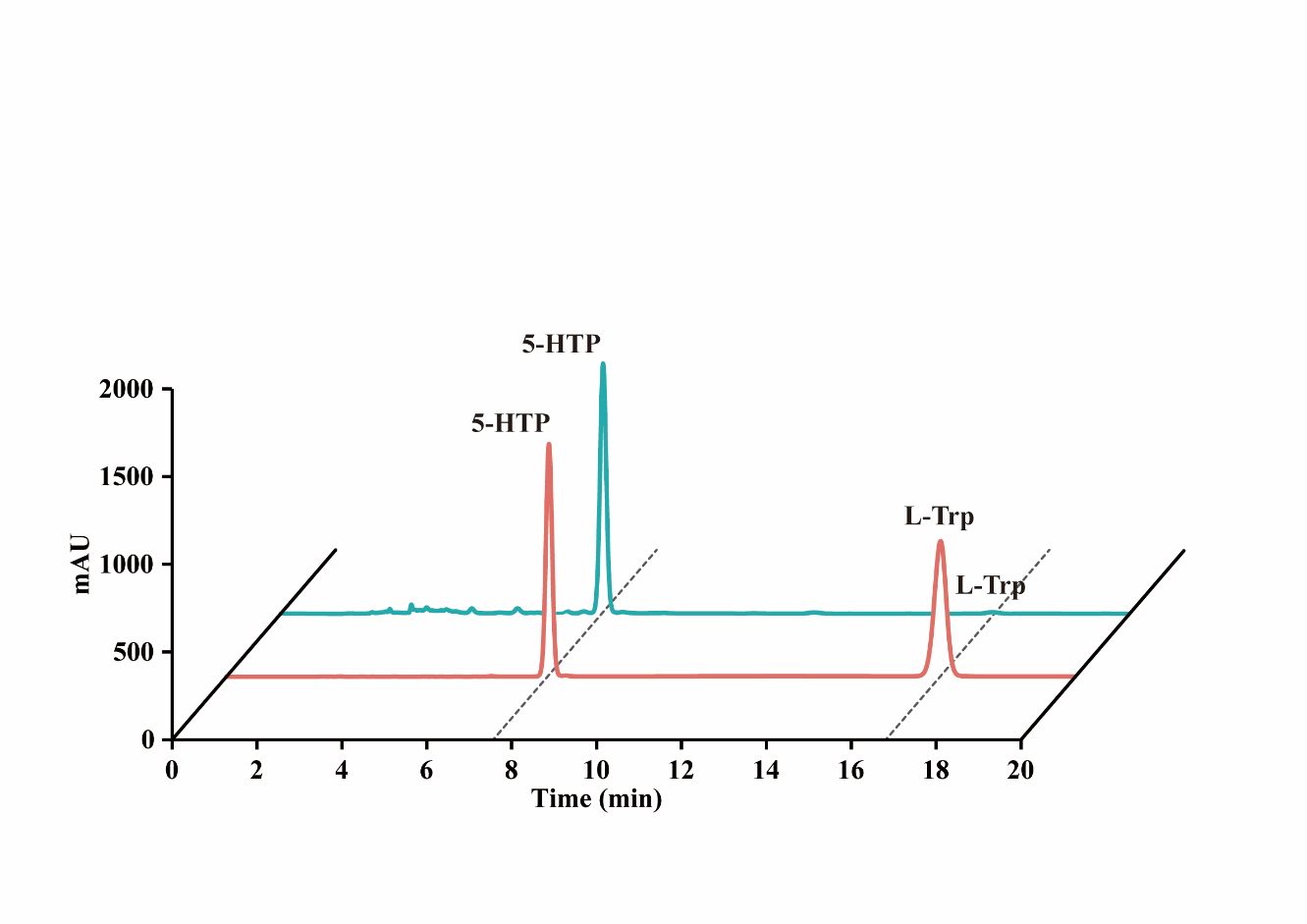


Fig. S6 HPLC detection information of 5-HTP and L-Trp

Red curve represents the detection with a 5-HTP standard (0.5 g/L) and green curve represents the detection result of the fermentation broth of engineered strain HTP11 with 15× fold dilution. The retention time was 7.5 min for 5-HTP and 16.8 min for L-Trp.


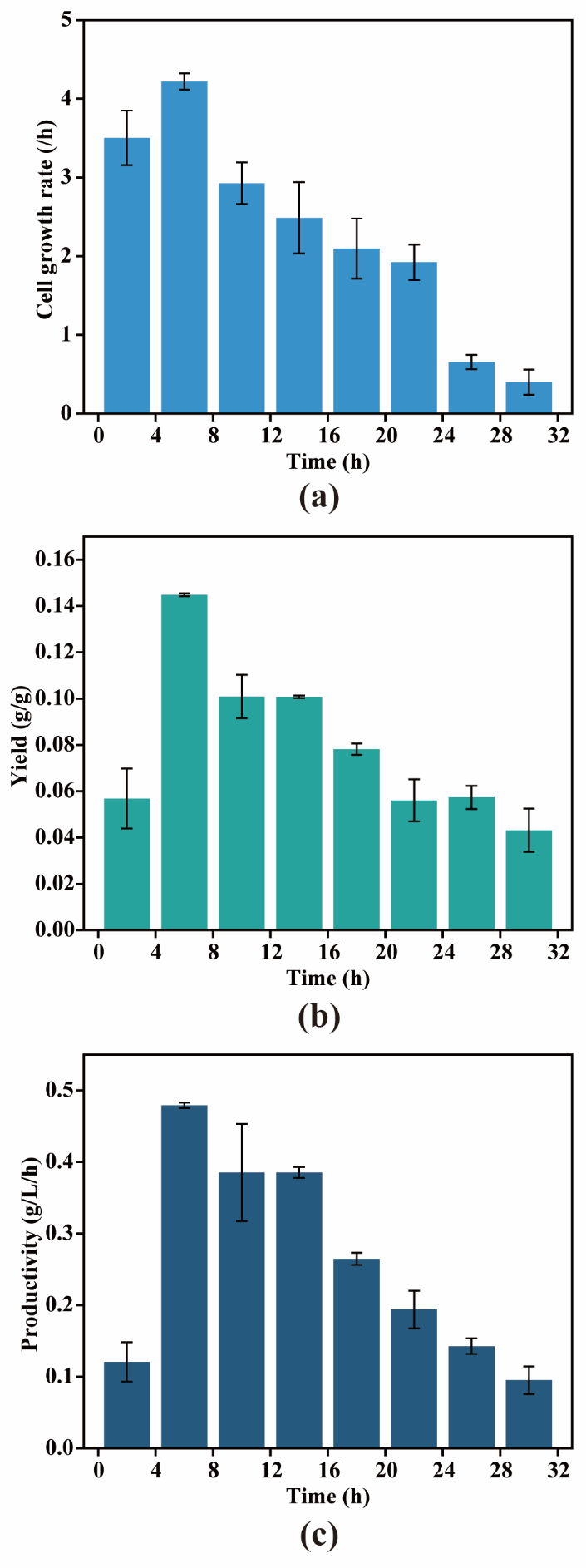


Fig. S7 Real-time efficiency of 5-HTP production by HTP11 in a 5L bioreactor. (a) Cell growth rate. (b) Yield. (c) Productivity. The data are presented as averages, and the error bars represent standard deviations (n = 3 independent experiments).


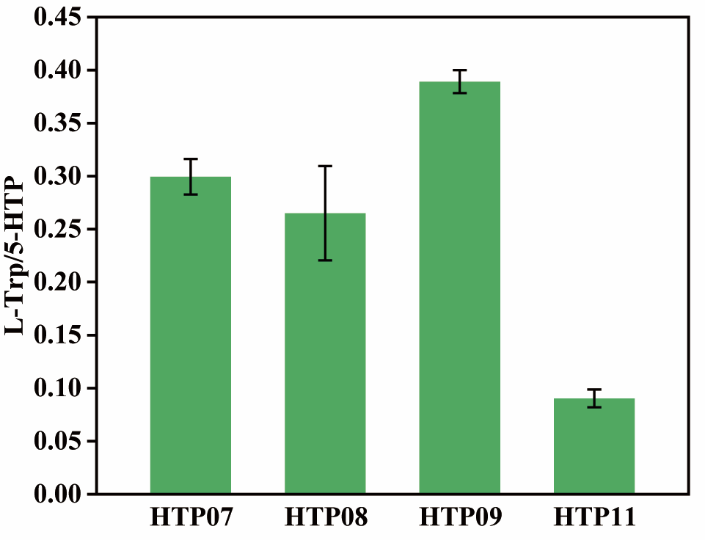


Fig. S8 The ratio of tryptophan to 5-HTP in shake flask fermentation results of HTP07-09 and 10 strains.


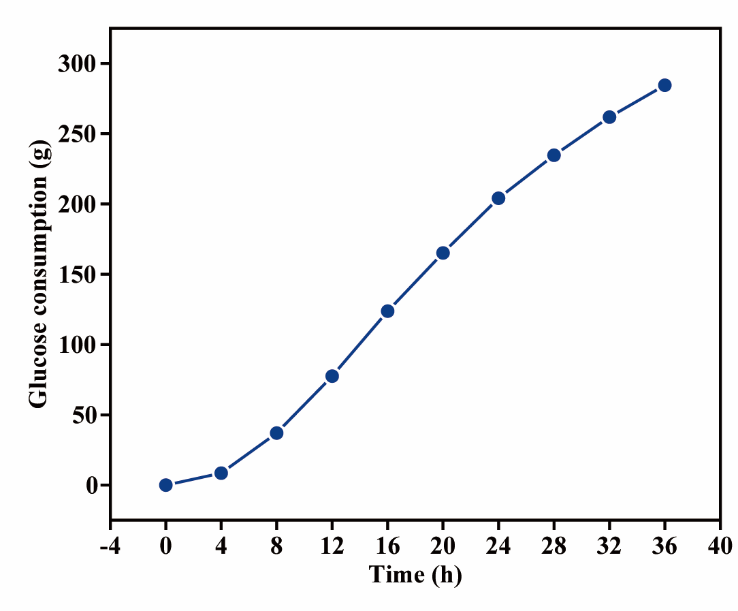


Fig. S9 Glucose consumption of HTP11 strain in a 5-L bioreactor

Tab. S2 The synthesized heterologous protein sequence applied in this study.

| **Protein** | **Protein Sequence** |
| --- | --- |
| GCHⅠ | MKEVNKEQIEQAVRQILEAIGEDPNREGLLDTPKRVAKMYAEVFSGLNEDPKEHFQTIFGENHEELVLVKDIAFHSMCEHHLVPFYGKAHVAYIPRGGKVTGLSKLARAVEAVAKRPQLQERITSTIAESIVETLDPHGVMVVVEAEHMCMTMRGVRKPGAKTVTSAVRGVFKDDAAARAEVLEHIKRQD |
| PTPS | MNAAVGLRRRARLSRLVSFSASHRLHSPSLSAEENLKVFGKCNNPNGHGHNYKVVVTIHGEIDPVTGMVMNLTDLKEYMEEAIMKPLDHKNLDLDVPYFADVVSTTENVAVYIWENLQRLLPVGALYKVKVYETDNNIVVYKGE |
| SPR | MEGGRLGCAVCVLTGASRGFGRALAPQLAGLLSPGSVLLLSARSDSMLRQLKEELCTQQPGLQVVLAAADLGTESGVQQLLSAVRELPRPERLQRLLLINNAGTLGDVSKGFLNINDLAEVNNYWALNLTSMLCLTTGTLNAFSNSPGLSKTVVNISSLCALQPFKGWGLYCAGKAARDMLYQVLAVEEPSVRVLSYAPGPLDTNMQQLARETSMDPELRSRLQKLNSEGELVDCGTSAQKLLSLLQRDTFQSGAHVDFYDI |
| PCD | MAGKAHRLSAEERDQLLPNLRAVGWNELEGRDAIFKQFHFKDFNRAFGFMTRVALQAEKLDHHPEWFNVYNKVHITLSTHECAGLSERDINLASFIEQVAVSMT |
| DHPR | MAAGEARRVLVYGGRGALGSRCVQAFRARNWWVASVDVVENEEASASIIVKMTDSFTEQADQVTAEVGKLLGEEKVDAILCVAGGWAGGNAKSKSLFKNCDLMWKQSIWTSTISSHLATKHLKEGGLLTLAGAKAALDGTPGMIGYGMAKGAVHQLCQSLAGKNSGMPPGAAAIAVLPVTLDTPMNRKSMPEADFSSWTPLEFLVETFHDWITGKNRPSSGSLIQVVTTEGRTELTPAYF |
| TM | MELEDVPWFPRKISELDKCSHRVLMYGSELDADHPGFKDNVYRQRRKYFVDVAMGYKYGQPIPRVEYTEEETKTWGVVFRELSKLYPTHACREYLKNFPLLTKYCGYREDNVPQLEDVSMFLKERSGFTVRPVAGYLSPRDFLAGLAYRVFHCTQYIRHGSDPLYTPEPDTCHELLGHVPLLADPKFAQFSQEIGLASLGASDEDVQKLATCYFFTIEFGLCKQEGQLRAYGAGLLSSIGELKHALSDKACVKAFDPKTTCLQECLITTFQEAYFVSESFEEAKEKMRDFAKSITRPFSVYFNPYTQSIEILKDT |
| TM1 | MKLEDVPWFPRKISELDKCSHRVLMYGSELDADHPGFKDNVYRQRRKYFVDVAMGYKYGQPIPRVEYTEEETKTWGVVFRELSKLYPTHACREYLKNFPLLTKYCGYREDNVPQLEDVSMFLKERSGFTVRPVAGYLSPRDFLAGLAYRVFHCTQYIRHGSDPLYTPEPDTCHELLGHVPLLADPKFAQFSQEIGLASLGASDEDVQKLATCYFFTIEFGLCKQEGQLRAYGAGLLSSIGELKHALSDKACVKAFDPKTTCLQECLITTFQEAYFVSESFEEAKEKMRDFAKSITRPFSVYFNPYTQSIEILKDT |
| TM2 | MKLEDVPWFPRKISELDKCSHRVLMYGSELDADHPGFKDNVYRQRRKYFVDVAMGYKYGQPIPRVEYTEEETKTWGVVFRELSKLYPTHACREYLKIFCLLTKYCGYREDNVPQLEDVSMFLKERSGFTVRPVAGYLSPRDFLAGLAYRVFHCTQYIRHGSDPLYTPEPDTCHELLGHVPLLADPKFAQFSQEIGLASLGASDEDVQKLATCYFFTIEFGLCKQEGQLRAYGAGLLSSIGELKHALSDKACVKAFDPKTTCLQECLITTFQEAYFVSESFEEAKEKMRDFAKSITRPFSVYFNPYTQSIEILKDT |
| GDH*_esi_* | MGYNSLKGKVAIVTGGSMGIGEAIIRRYAEEGMRVVINYRSHPEEAKKIAEDIKQAGGEALTVQGDVSKEEDMINLVKQTVDHFGQLDVFVNNAGVEMPSPSHEMSLEDWQKVIDVNLTGAFLGAREALKYFVEHNVKGNIINMSSVHEIIPWPTFVHYAASKGGVKLMTQTLAMEYAPKGIRINAIGPGAINTPINAEKFEDPKQRADVESMIPMGNIGKPEEISAVAAWLASDEASYVTGITLFADGGMTLYPSFQAGRG |

1. Agus A, Planchais J, Sokol H: Gut microbiota regulation of tryptophan metabolism in health and disease. *Cell host & Microbe* 2018, 23:6.
2. Hinz M, Stein A, Uncini T: 5-HTP efficacy and contraindications. *Neuropsychiatric disease and treatment* 2012, 8:323-328.
3. Sun XX, Lin YH, Yuan QP, Yan YJ: Precursor-directed biosynthesis of 5-hydroxytryptophan using metabolically engineered *E. coli*. *Acs Synthetic Biology* 2014, 4(5):554-558.
4. Lin YH, Sun XX, Yuan QP, Yan YJ: Engineering bacterial phenylalanine 4-hydroxylase for microbial synthesis of human neurotransmitter precursor 5-hydroxytryptophan. *Acs Synthetic Biology* 2014, 3(7):497-505.
5. Mora-Villalobos JA, Zeng AP: Synthetic pathways and processes for effective production of 5-hydroxytryptophan and serotonin from glucose in *Escherichia coli*. J*ournal of Biological Engineering* 2018, 12:3.
6. Hara R, Kino K: Enhanced synthesis of 5-hydroxy-L-tryptophan through tetrahydropterin regeneration. *AMB Express* 2013, 3:70.
7. Wang HJ, Liu WQ, Feng S, Lei H, Lian JH, Liang Q, Jin C, Xu ZN: Metabolic pathway engineering for high-level production of 5-hydroxytryptophan in *Escherichia coli*. *Metabolic Engineering* 2018, 48:279-287.
